# UBE3A expression during early postnatal brain development is required for proper dorsomedial striatal maturation

**DOI:** 10.1172/jci.insight.166073

**Published:** 2023-02-22

**Authors:** Diana C. Rotaru, Ilse Wallaard, Maud de Vries, Julia van der Bie, Ype Elgersma

**Affiliations:** 1 Department of Clinical Genetics and; 2 ENCORE Expertise Center for Neurodevelopmental Disorders, Erasmus MC, Rotterdam, Netherlands.

**Keywords:** Cell Biology, Mouse models, Neurodevelopment, Neurological disorders

## Abstract

Angelman syndrome (AS) is a severe neurodevelopmental disorder (NDD) caused by loss of functional ubiquitin protein ligase E3A (UBE3A). Previous studies showed that UBE3A plays an important role in the first postnatal weeks of mouse brain development, but its precise role is unknown. Since impaired striatal maturation has been implicated in several mouse models for NDDs, we studied the importance of UBE3A in striatal maturation. We used inducible *Ube3a* mouse models to investigate the maturation of medium spiny neurons (MSNs) from dorsomedial striatum. MSNs of mutant mice matured properly till postnatal day 15 (P15) but remained hyperexcitable with fewer excitatory synaptic events at later ages, indicative of stalled striatal maturation in *Ube3a* mice. Reinstatement of UBE3A expression at P21 fully restored MSN excitability but only partially restored synaptic transmission and the operant conditioning behavioral phenotype. Gene reinstatement at P70 failed to rescue both electrophysiological and behavioral phenotypes. In contrast, deletion of *Ube3a* after normal brain development did not result in these electrophysiological and behavioral phenotypes. This study emphasizes the role of UBE3A in striatal maturation and the importance of early postnatal reinstatement of UBE3A expression to obtain a full rescue of behavioral phenotypes associated with striatal function in AS.

## Graphical abstract

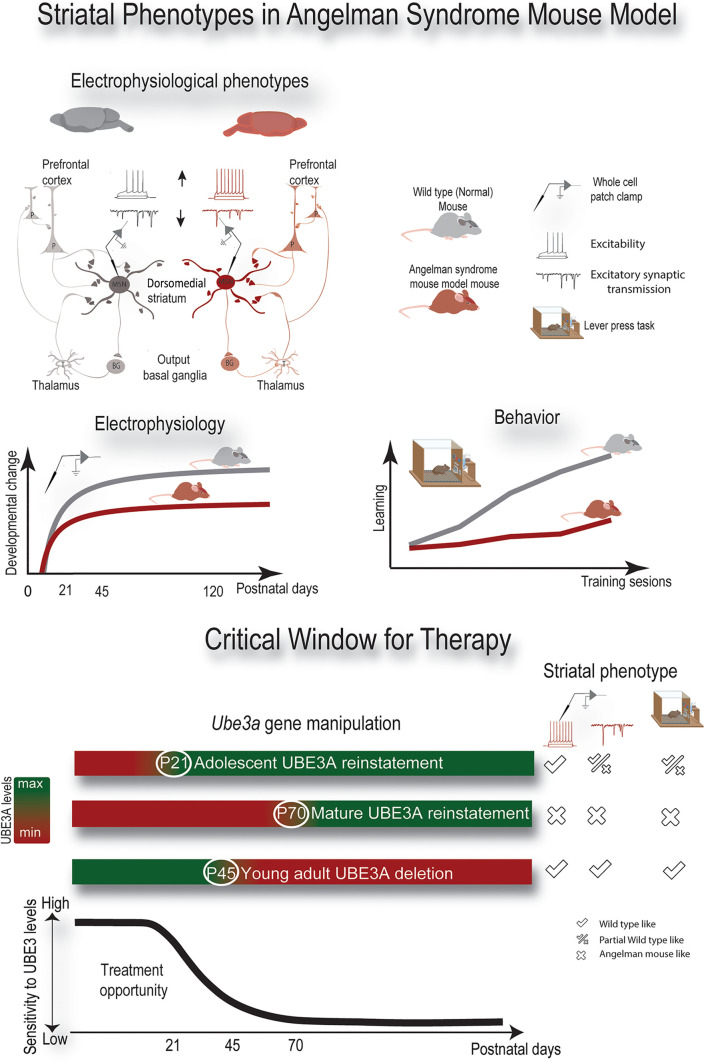

## Introduction

Neurodevelopmental disorders (NDDs) are brain diseases caused by heterogenous genetic lesions that often generate overlapping clinical phenotypes, including intellectual disability, autism spectrum disorder (ASD), motor dysfunction, and epilepsy (1, 2). Such phenotypes emerge early postnatally and progress toward increased severity, pointing to defects of neuronal circuit maturation (3). Neuronal circuits mature during critical periods, beyond which it becomes difficult to modify them (4, 5). Delayed or faster closing of critical periods has long-lasting changes at the synaptic level (6–10). Genetic treatments represent an increasingly used strategy for NDDs, yet such approaches may not achieve a brain-wide spread of the drugs delivered. Thus, it is important to identify both the neuronal circuits that underlie the clinical phenotypes and their critical window for therapeutic intervention. Increasing evidence implicates basal ganglia (BG) dysfunction in many NDDs with specific importance for the motor phenotypes (11, 12). Here we focused on understanding the maturation profile of the striatum, the input segment of the BG, using our inducible mouse model of Angelman syndrome (AS), a severe NDD with strong ASD features and motor dysfunction (13).

Loss of neuronal ubiquitin protein ligase E3A (UBE3A) results in AS, a severe NDD characterized by intellectual disability, absence of speech, gross and fine motor deficits, behavioral abnormalities, and often epilepsy (13). Clinical or genetic diagnosis of AS is typically provided after the first year, as the manifestation of impaired neurodevelopment is not immediately apparent (14). Assessment of global development shows that individuals with AS make slow gains up to approximately 12 years of age at about 1–2 months per year based on age-equivalent scores (15), indicating impaired postnatal maturation of specific brain circuits (7, 16). Understanding the neuronal maturation process in AS may provide mechanistic insight into the disease pathophysiology and may guide us toward identifying the optimal time point for initiating gene-based therapies (17–19).

Impaired fine and gross motor dysfunction and lack of speech are distinct clinical phenotypes in individuals with AS (15) and presented in the first description of the syndrome by Harry Angelman (20). The precise neuronal circuits underlying these motor phenotypes is unknown. Studies in mouse models indicate that a role of the cerebellum in the motor deficits in AS mice is unlikely (21). Alternatively, the motor phenotypes are potentially associated with BG dysfunction (22). BG is a region of the brain that integrates sensory inputs with internal drives to generate purposeful motor action (23, 24). Further support for the involvement of the BG comes from imaging studies showing that its gray matter and functional connectivity with the cortex and thalamus are decreased in individuals with AS (25, 26). Moreover, mouse models show deficits in the lever press (“operant conditioning”) task (27). This indicates that the dorsomedial striatum (DMS), the medial input segment of BG, is dysfunctional in AS (28). Additionally, the medium spiny neurons (MSNs), the vast majority of neurons in the striatum, show electrophysiological changes in mouse models of AS (27). The striatum is divided into 3 functional territories: the DMS “cognitive” region, the dorsolateral striatum “motor” region, and the ventral “limbic” region incorporating the nucleus accumbens (29–31). AS mouse models point to differences between the striatal regions, regarding either dopamine levels or synaptic transmission (27, 32, 33). Here we focused on the DMS for several reasons: 1) the DMS is involved in the early phases of motor learning (involving multiple “cognitive” aspects) (34) and motor learning deficits, which are clearly present both in patients and in AS mouse models (35); 2) our previous data showed that neurons in layer 5 of the prefrontal cortex (PFC) from AS mice have increased excitation-to-inhibition ratio (36, 37), and the activity of layer 5 PFC pyramidal neurons affects the maturation of MSNs from the DMS via their glutamatergic inputs onto MSNs (38); and 3) previous data showed changes in the electrophysiological properties of MSNs from the DMS (27).

Here, we used whole-cell patch clamp recordings, to determine how lack of UBE3A affects the maturation of MSNs of the DMS during development. Additionally, we used the lever press task to validate previously identified deficits in operant learning of AS mice. Furthermore, using inducible *Ube3a* mice, we identified the critical window for rescuing the electrophysiological and the behavioral operant conditioning phenotypes.

## Results

### Absence of UBE3A generates increased firing rates and decreased excitatory transmission in MSNs from mature DMS.

**Figure 1:**
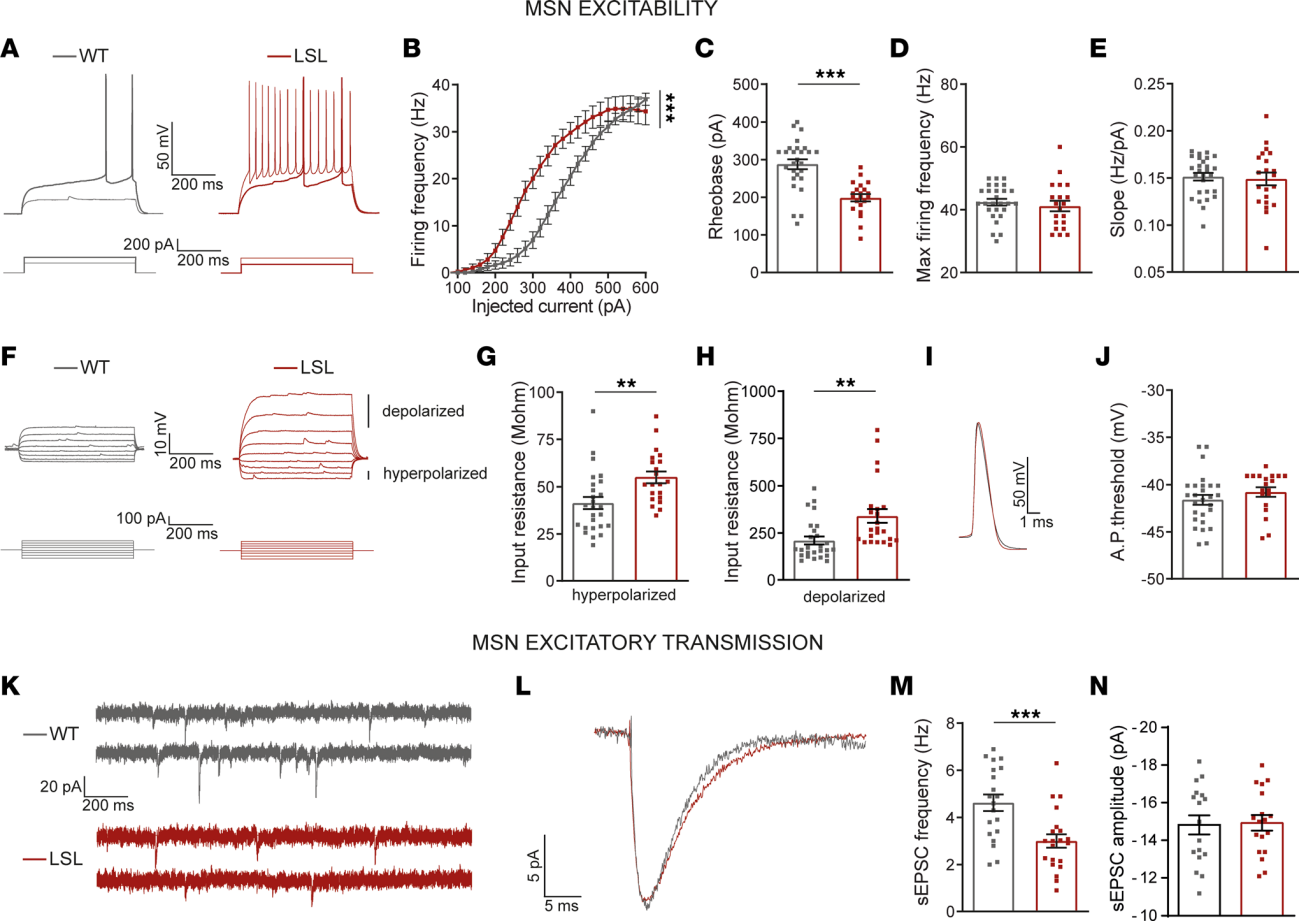
Absence of UBE3A generates increased firing rates and decreased excitatory transmission in MSNs from mature DMS. (**A**) Representative firing pattern of MSNs (top) obtained with current injection (bottom); thick traces represent the response to rheobase current. (**B**) F-I curves, 1-way repeated measures (RM) ANOVA (*F_1,49_* = 7.90, *P* = 0.0005). (**C**) Rheobase, 2-tailed unpaired *t* test (*t* = 5.17 df = 47, *P* = 0.0001). (**D**) Maximum firing rate, 2-tailed unpaired *t* test (*t* = 0.66 df = 47, *P* = 0.52). (**E**) F-I slope, 2-tailed unpaired *t* test (*t* = 0.33 df = 47, *P* = 0.75). (**F**) Representative voltage responses of MSNs (top), obtained by 30 pA current increments between –100 pA and +140 pA (bottom). (**G**) Input resistance at hyperpolarized domain, 2-tailed unpaired *t* test (*t* = 3.16 df = 47, *P* = 0.0028). (**H**) Input resistance at depolarized domain, 2-tailed unpaired *t* test (*t* = 3.03 df = 47 *P* = 0.0041). (**I**) Examples of single AP. (**J**) AP threshold, 2-tailed unpaired *t* test (*t* = 1.12 df = 47, *P* = 0.26). (**K**) Representative recordings of sEPSCs from MSNs. (**L**) Representative average sEPSCs. (**M**) sEPSC frequency, 2-tailed unpaired *t* test (*t* = 3.63 df = 39, *P* = 0.0008). (**N**) sEPSC amplitude, 2-tailed unpaired *t* test (*t* = 0.17 df = 39, *P* = 0.87). Sample size (*N* = neurons/mice) for **B**–**E** and **G**–**J**: WT: *N* = 27/7; LSL: *N* = 21/5 and for **M** and **N**: WT: *N* = 20/7; LSL: *N* = 21/5. Data represented as dot plots (1 neuron) with mean ± SEM. **P ≤ 0.01, ***P ≤ 0.001. See also Supplemental Table 1.

To identify how absence of UBE3A changes the electrophysiological properties of MSNs, we performed whole-cell current clamp recordings from MSNs in striatal slices from adult (P110–P175) *Ube3a^mStop/p+^* (LSL) and *Ube3a^m+/p+^* wild-type (WT) littermates. Upon increasing depolarization current, we observed a significant left shift in the average firing frequency versus injected current (F-I) relationship between MSNs from LSL and WT mice (Figure 1, A and B), pointing to increased excitability in MSNs from LSL mice. The rheobase representing the minimum current that triggered at least 1 single action potential (AP) was significantly lower in LSL MSNs than WT (Figure 1, A and C). Values of rheobase of about 30% lower in LSL than WT were sufficient to trigger APs in LSL MSNs (Supplemental Table 1; supplemental material available online with this article; https://doi.org/10.1172/jci.insight.166073DS1; P130).

Next, we asked whether the changes in rheobase reflected changes in active, passive, or both properties of MSNs. To investigate the active properties that are dependent on voltage-gated ion channels, we focused on several parameters, including maximum firing rates, the F-I slope, and the AP firing threshold. We found no differences in any of these parameters between LSL and WT mice, including the maximum firing rate (Figure 1D), F-I slope (Figure 1E), and AP firing threshold (Figure 1, I and J, and Supplemental Table 2, P130). To investigate the passive properties, we analyzed the input resistance of MSNs. In MSNs, the input resistance is mostly dependent on inwardly rectifying potassium channels (Kirs) at hyperpolarized potentials, while the leak potassium channels set the input resistance at depolarized potentials (39–41). We thus calculated the input resistance of MSNs on both the hyperpolarized (Figure 1, F and G) and the depolarized (Figure 1, F and H) voltage responses to current injections. Note that for the same neuron, the hyperpolarizing voltages are smaller than the depolarizing ones, pointing to different ion channels for the 2 domains. We found that input resistance was significantly larger in MSNs from LSL than WT mice for both the nonrectified (hyperpolarized) and rectified (depolarized) domains. In the depolarized domain the input resistance was more than 50% higher in LSL than WT mice (Supplemental Table 1), matching the rheobase difference mentioned above. In the hyperpolarized domain, the input resistance was only about 30% higher in LSL than WT mice (Supplemental Table 1). These data suggest that the increased firing rates observed in MSNs from LSL mice are likely due to an increased input resistance in these cells. Thus, upon similar depolarizing current injection into LSL and WT MSNs, the higher input resistance in LSL MSNs leads to higher voltage responses than WT, further triggering stronger activation of sodium channels and increased number of APs. To test if the input resistance fully accounts for the increased firing rates, we next asked whether equivalent neuronal depolarization between these WT and LSL mice also produced a difference in firing rate. We measured the membrane potential at 25 ms after depolarizing current injection and counted the number of APs that followed this depolarization. We found that similar levels of depolarization generated similar numbers of APs in both WT and LSL MSNs (Supplemental Figure 1, A and B). These data are also in line with the lack of differences in AP threshold of MSNs from the 2 genotypes (Figure 1, I and J).

Next, we investigated whether absence of UBE3A affects excitatory postsynaptic currents of MSNs. We voltage-clamped the MSNs from DMS at –70 mV, to record spontaneous excitatory postsynaptic currents (sEPSCs) (Figure 1K). LSL mice had significantly lower sEPSCs frequency (Figure 1, K and M) without a change in amplitude (Figure 1, L and N). The sEPSC frequency was about 30% lower in LSL than WT mice (Figure 1, K and M, and Supplemental Table 1), but the sEPSC amplitude was similar between genotypes (Figure 1, L and N, WT: –14.83 ± 0.52 pA; LSL: –15.00 ± 0.41 pA). Because sEPSCs represent a mixture of AP-mediated excitatory events and single synapse–mediated events (37, 42), we next investigated if miniature EPSCs (mEPSCs) are also affected by the loss of UBE3A proteins in MSNs. We voltage-clamped the MSNs at –70 mV in the presence of tetrodotoxin to block AP-mediated glutamate release (Supplemental Figure 1C). LSL mice had significantly lower mEPSC frequency (Supplemental Figure 1E, WT: 3.10 ± 0.27 Hz; LSL: 2.08 ± 0.17 Hz) without a change in amplitude (Supplemental Figure 1, D and F, WT: –13.58 ± 0.43 pA; LSL: –12.54 ± 0.46 pA). These data suggest that the changes in synaptic transmission may result from fewer functional synapses on MSNs from LSL mice.

Together, these findings indicate that absence of UBE3A enhances the intrinsic excitability and decreases the frequency of sEPSCs in MSNs from DMS, which from now on we will refer to as electrophysiological phenotypes.

### Absence of UBE3A leads to a stalled striatal neurodevelopment.

**Figure 2:**
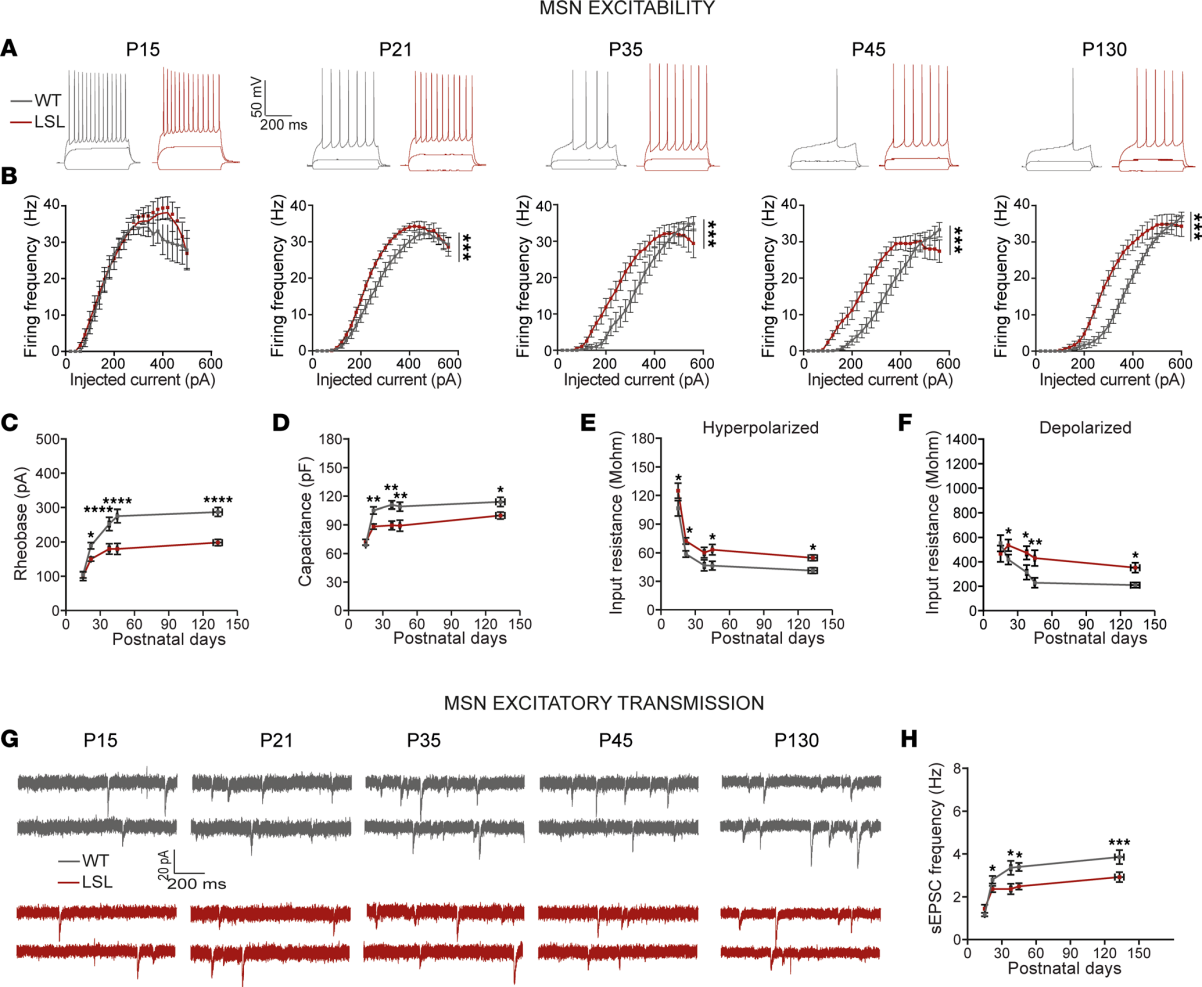
Absence of UBE3A leads to stalled striatal neurodevelopment. (**A**) Representative voltage responses at indicated postnatal days obtained with 3 current injections: 1) hyperpolarizing (–100 pA), 2) subthreshold depolarizing (+50 pA), and 3) over-threshold depolarizing (+250 pA) currents showing changes of input resistance at hyperpolarized and depolarized domains, and of firing rates, respectively. (**B**) F-I curves, 1-way RM ANOVA (P15: *F*_1,34_ = 0.28, *P* = 1.00; P21: *F*_1,50_ = 2.36, *P* = 0.0005; P35: *F*_1,34_ = 2.70, *P* = 0.0005; P45: *F*_1,50_ = 4.65, *P* = 0.0005; P130: *F*_1,49_ = 7.90, *P* = 0.0005). (**C**) Rheobase, factorial ANOVA (*F*_4,231_ = 3.42, *P* = 0.01, see Supplemental Table 1). (**D**) Capacitance, factorial ANOVA (*F*_4,231_ = 1.73, *P* = 0.14), with age (*F*_4,231_ = 14.74, *P* = 0.0001) and genotype (*F*_1,231_ = 23.611, *P* = 0.0001). (See Supplemental Table 1.) (**E** and **F**) Input resistance at depolarized (**E**) and hyperpolarized (**F**) domains, factorial ANOVA (in **E**, *F*_4,231_ = 1.64, *P* = 0.165; and in **F**, *F*_4,231_ = 0.09, *P* = 0.984), with age (in **E**, *F*_4,231_ = 7.156, *P* = 0.0001; and in **F**, *F*_4,231_ = 48.188, *P* = 0.0001) and genotype (in **E**, *F*_1,231_ = 10.363, *P* = 0.0001; and in **F**, *F*_1,231_ = 23.187, *P* = 0.0001). (See Supplemental Table 1.) (**G**) Representative recordings of sEPSCs at indicated postnatal days. (**H**) sEPSC frequency, factorial ANOVA (*F*_4,205_ = 2.87, *P* = 0.024). Sample size (*N* = neurons/mice) (P15: WT: *N* = 13/2; LSL: *N* = 13/2; P21: WT: *N* = 37/5; LSL: *N* = 38/5; P35: WT: *N* = 17/3; LSL: *N* = 13/2; P45: WT: *N* = 21/3; LSL: *N* = 26/4; P130: WT: *N* = 27/7; LSL: *N* = 21/5). Data represent mean ± SEM. *P ≤ 0.05, **P ≤ 0.01, ***P ≤ 0.001, ****P ≤ 0.0001. See also Supplemental Table 1.

**Figure 3:**
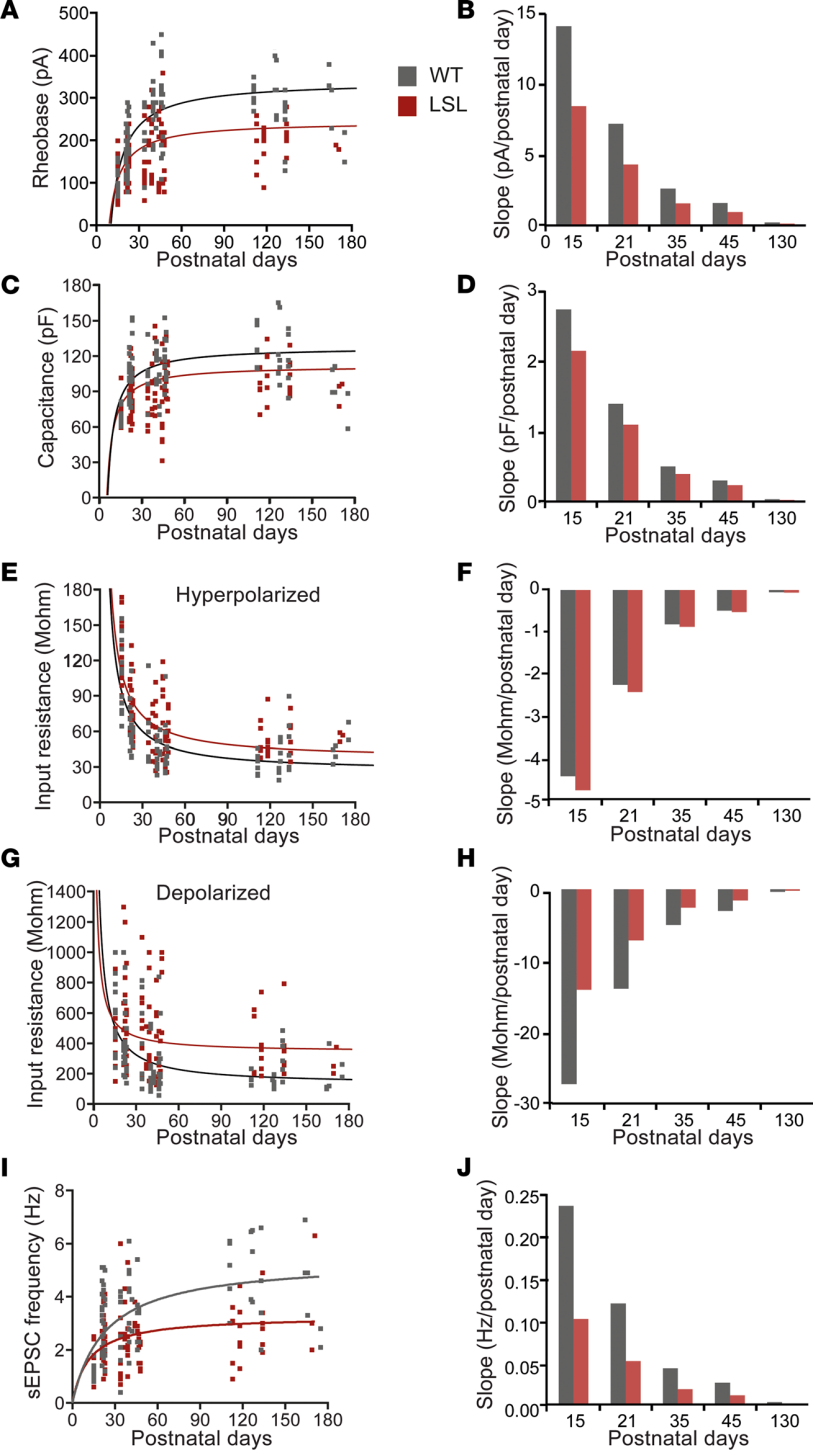
Inverse function model indicates a slower development of MSNs in the absence of UBE3A. (**A**, **C**, **E**, **G**, and **I**) Inverse function [*F(postnatal day) = B/postnatal day + constant*] regression analysis on individual values for rheobase (**A**), capacitance (**C**), input resistance hyperpolarized (**E**) and depolarized (**G**), and sEPSC frequency (**I**) against postnatal days; the lines illustrate severe delay of the MSNs from LSL versus WT mice (see Table 1 for specific values of obtained parameters). (**B**, **D**, **F**, **H**, and **J**) Slope (first derivative of the inverse function) at different postnatal day (P15, P21, P35, P45, P130) illustrates that LSL have a slower developmental speed than WT for rheobase (**B**), capacitance (**D**), input resistance depolarized (**H**), and sEPSC frequency (**J**) but similar speed for input resistance hyperpolarized (**F**) (see Table 1 for specific values). Data represented as dot plots (1 neuron).

In rodents, the striatum matures during the first 4 weeks postnatal (43–45). To identify when the electrophysiological phenotypes emerge, we obtained a developmental profile of the electrophysiological properties by performing whole-cell patch clamp experiments from MSNs in the DMS between P15 and P175, in WT and LSL mice (Figures 2 and 3). For each electrophysiological parameter, we analyzed the data both by age groups (Figure 2) and by performing a regression analysis with curve fitting (see Methods, Supplemental Table 2, Figure 3, and Table 1) (46). The following age groups were analyzed: P15 (data collected at P15 only), P21 (data collected between P21 and P23), P35 (data collected between P34 and P40), P45 (data collected between P42 and P48), and P130 (data collected between P110 and P175).

The F-I relationship between MSNs from LSL and WT was similar at P15 but started to left shift at P21 and became significantly different at this time point (Figure 2, A and B). In line with the F-I left shift, the rheobase was significantly lower in MSNs from LSL than WT mice starting also from P21 (Figure 2C). Moreover, the rheobase values were differently affected by neurodevelopment in the LSL compared with WT (Figure 2C). Thus, the rheobase was similar between the LSL and WT mice at P15 and dropped by about 21% at P21, followed by a 28% drop at P35 and finally by a 34% drop at P45, remaining at similar values at P130 (see Supplemental Table 1). Next, we calculated the capacitance and input resistance (at both hyperpolarized and depolarized domains), because these properties strongly affect the firing rates of neurons (39–41). As expected, the capacitance increased, while the input resistance decreased over time, and both parameters were significantly different in MSNs from LSL than WT mice starting with P21 (Figure 2, D–F). Thus, the capacitance remained about 12-20% lower starting at P21 (see Supplemental Table 1). The input resistance decreased over time in both domains, but starting from P21, it remained consistently higher in the MSNs from LSL than WT mice. Unlike the capacitance, the magnitude of change in input resistance increased over time, especially in the depolarized domain, from about 27% at P21, to 50% at P35, 88% at P45,and 68% at P130 (see Supplemental Table 2).

In contrast to the passive properties, the F-I slope, maximum firing rate and AP threshold remained unchanged between genotypes (Supplemental Figure 2, A–C), though these measures changed over time (see Supplemental Table 2). These data suggest that the hyperexcitability phenotype is very likely the result of changes in passive properties, including the decreased capacitance and increased membrane resistance.

To find when changes in synaptic transmission start to appear, we voltage-clamped MSNs at –70 mV to record sEPSCs (Figure 2G). Similar to the changes in excitability, the LSL mice had a significantly lower sEPSC frequency starting at P21 that increased over time (Figure 2H and Supplemental Table 1).

Collectively, these results show that at P15, MSNs of both LSL and WT mice have similar electrophysiological phenotypes, and differences between genotypes emerge at P21.

### Inverse function model indicates a slower development of MSNs in the absence of UBE3A.

To obtain a better prediction of the electrophysiological changes during development, we performed an exploratory regression analysis with curve fitting to identify the model that fit our data best using the IBM Statistical Package for the Social Sciences (SPSS) curve estimation function (46). Although multiple curves were found to significantly fit the data, the *F* values for each model (Supplemental Table 2) suggested that the best fit for the rheobase, capacitance, input resistance, and sEPSC frequency was an “inverse function” [F(postnatal day) = B*(1/postnatal day) + Constant]. In contrast, the rest of the parameters, including the F-I slope, maximum firing frequency, and AP threshold, were not well fit by any of the tested models (Supplemental Table 3).

We compared the inverse function [F(postnatal day) = B*(1/postnatal day) + Constant] fit for LSL versus WT (Figure 3) by obtaining the values of *B* and *Constant* for rheobase, capacitance, input resistance (hyperpolarized and depolarized), and sEPSC frequency (Table 1). The values of the coefficients showed strong differences between LSL ad WT for most of the tested parameters, pointing to an overall slower development of LSL mice (values of the *B* parameter) and lower “steady-state mature” (values of the *Constant*) (Figure 3, A, C, E, G, and I, and Table 1). Next, we asked if indeed the rate of maturation is different between LSL and WT mice. To answer this question, we calculated the slope at specific postnatal days (P15, P21, P35, P45, P130) by calculating the first derivative for each parameter (Figure 3, B, D, E, H, and J, and Table 1). For both genotypes the model predicted that the rheobase, a measure of excitability, has the fastest increase at P15 and becomes gradually slower close to an asymptotic value at P130. Nevertheless, the slope of the rheobase was slower in LSL than WT mice at all the postnatal time points (Figure 3B and Table 1). These values suggest that the MSN excitability in LSL mice is maturing slower than WT. In line with this idea, both the capacitance (Figure 3D and Table 1) and the input resistance in the depolarized domain (Figure 3H and Table 1) showed the fastest change at P15 for both genotypes, but the development was slower in LSL than WT mice. An exception to this pattern is the input resistance in the hyperpolarized domains, which developed at a similar speed between LSL and WT, even though the LSL mice had decreased overall levels of input resistance at all ages (Figure 2, E and F, and Table 1). Similar to the excitability data, the sEPSC frequency, which is a measure of the excitatory synaptic transmission, also showed reduced increase over time in LSL compared with WT. For all parameters the rate of development was close to the asymptotic level (almost 0) in adulthood. This model suggests that the rate of change for the majority of the MSN electrophysiological parameters is the fastest between P15 and P21 for both genotypes, yet the LSL mice have an overall slower maturation than WT.

### A critical window for reversal of the striatal electrophysiological deficits.

**Figure 4:**
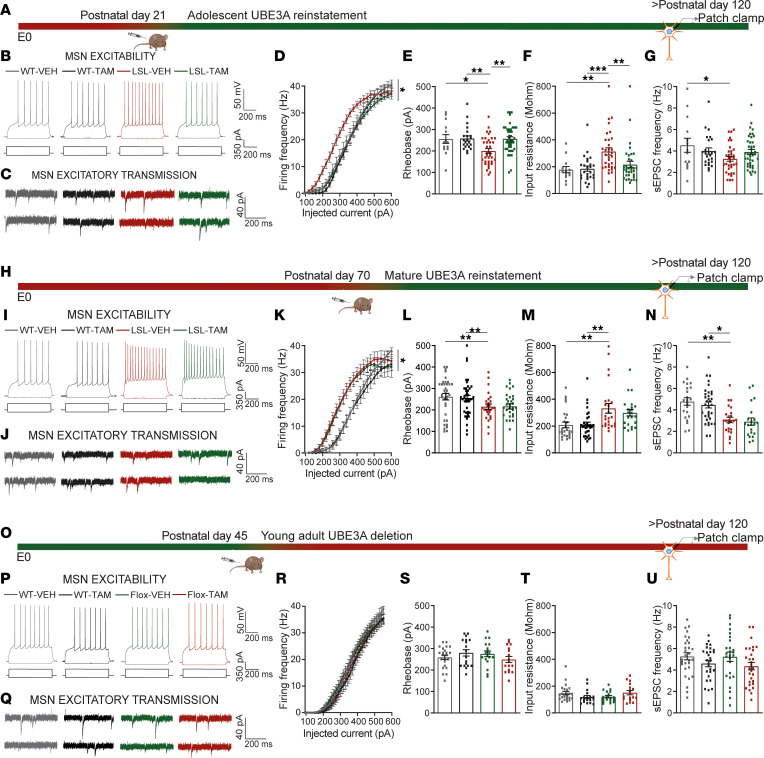
A critical window for rescuing the striatal electrophysiological deficits. (**A**, **H**, and **O**) Schematic of *Ube3a* gene reinstatement (**A** and **H**) or deletion (**O**) at indicated postnatal days and time point of electrophysiological recordings. (**B**, **I**, and **P**) Representative voltage responses from MSNs (top), obtained with hyperpolarizing (–100 pA) and depolarizing currents (350 pA) (bottom). (**D**, **K**, and **R**) F-I curves, 2-way RM ANOVA: (**D**) (*F*_1,108_ = 5.57, *P* = 0.02); post hoc Bonferroni’s: LSL-VEH against WT-VEH (*P* = 0.046), LSL-VEH against WT-TAM (*P* = 0.001), and LSL-VEH against LSL-TAM (*P* = 0.0001); (**K**) (*F*_1,67_ = 1.89, *P* = 0.92), with genotype effect; post hoc Bonferroni’s: LSL-VEH against WT-VEH (*P* = 0.016), LSL-VEH against WT-TAM (*P* = 0.005), and LSL-VEH against LSL-TAM (*P* = 1); (**R**) (*F*_1,69_ = 0.038, *P* = 0.5). (**E**, **L**, and **S**) Rheobase, 2-way ANOVA and post hoc Bonferroni’s (Supplemental Table 4): (**E**) (*F*_1,108_ = 4.17, *P* = 0.044); (**L**) (*F*_1,67_ = 0.12, *P* = 0.76, with genotype effect; (**S**) (*F*_1,69_ = 0.59, *P* = 0.49). (**F**, **M**, and **T**) Input resistance, 2-way ANOVA and post hoc Bonferroni’s (Supplemental Table 4): (**F**) (*F*_1,108_ = 6.27, *P* = 0.014); (**M**) (*F*_1,67_ = 0.04, *P* = 0.83), with genotype effect; (**T**) (*F*_1,69_ = 1.99, *P* = 0.16). (**C**, **J**, and **Q**) Representative recordings of sEPSCs from MSNs obtained by clamping the neurons at −70 mV. (**G**, **N**, and **U**) sEPSC frequency, 2-way ANOVA and post hoc Bonferroni’s (Supplemental Table 4): (**G**) (*F*_1,107_ = 5.16, *P* = 0.026); (**N**) (*F*_1,87_ = 0.22, *P* = 0.63), with genotype effect in (**U**) (*F*_1,69_ = 0.22, *P* = 0.63). Sample size (*N* = neurons/mice) (**D**–**G**) WT-VEH: *N* = 13/3; WT-TAM: *N* = 21/5; LSL-VEH, *N* = 33/5; LSL-TAM: *N* = 40/6); (**K**–**N**) WT-VEH: *N* = 17/5; WT-TAM: *N* = 15/3; LSL-VEH, *N* = 16/5; LSL-TAM: *N* = 17/5); (**R**–**U**) WT-VEH: *N* = 32/6; WT-TAM: *N* = 30/5; LSL-VEH, *N* = 22 /5; LSL-TAM: *N* = 24/6. Data represented as dot plots (1 neuron) with mean ± SEM. *P ≤ 0.05, **P ≤ 0.01, ***P ≤ 0.001.

Next, we aimed to determine the critical window for rescuing the striatal function by reinstating UBE3A expression. We showed previously that the rotarod deficit, a motor learning task strongly dependent on striatal function (47), is fully rescued if UBE3A expression is reinstated at P21 but not at P70 (19). Here we observed that the electrophysiological phenotype emerged around P21 and stabilized around P45. Thus, we hypothesized that the electrophysiological phenotype described above can be rescued at P21 but not at P70 *Ube3a* gene reinstatement. To test this hypothesis, we used our previously validated inducible AS mouse model Ube3a^mStop/p+^-Cre^Esr1*+^ (19, 37), in which UBE3A can be reinstated by tamoxifen-induced, Cre-mediated excision of the transcriptional stop cassette in the *Ube3a* gene. We treated the mice with either tamoxifen (LSL-TAM) or vehicle (LSL-VEH) at P21 or P70 and performed electrophysiological recordings after P120 (Figure 4, A and H). UBE3A levels in the striatum were restored to 83% of the WT levels in LSL-TAM mice upon TAM treatment in P21-treated pups and to 62% in P70 treated animals (Supplemental Figure 3B). The electrophysiological recordings showed that excitability was fully rescued at P21 reinstatement (Figure 4, A–G). In line with the significant interaction between genotypes (WT and LSL) and treatment revealed by factorial ANOVA, the F-I curves overlapped for the LSL-TAM and both WT-TAM and WT-VEH, while the LSL-VEH remained left shifted (Figure 4D). Rheobase and input resistance were both restored to WT levels in the LSL-TAM mice (Figure 4, E and F, and Supplemental Table 4). Synaptic transmission was only partially restored in LSL-TAM mice (Figure 4, C and G, and Supplemental Table 4). In contrast, P70 reinstatement failed to rescue both the excitability and the synaptic transmission phenotypes (Figure 4, H–N, and Supplemental Table 4). After P70 reinstatement, the F-I curves for the LSL-TAM mice overlapped with the LSL-VEH mice and were left shifted compared with WT-TAM and WT-VEH (Figure 4K and Supplemental Table 4). Additionally, the rheobase and input resistance failed to decrease (Figure 3, L and M), while the frequency of sEPSCs failed to increase in LSL-TAM mice (Figure 4, J and N, and Supplemental Table 4).

Because our data pointed to a mature state of MSNs around P45, we next aimed to determine whether UBE3A also plays a role in MSNs’ function after striatal development. For these experiments, we made use of the previously validated Ube3a^mFlox/p+^-Cre^Esr1*+^ (Flox-Cre) mice in which the *Ube3a* gene is efficiently deleted upon TAM injection (48, 49). We induced *Ube3a* gene deletion at P45 and performed the electrophysiological experiments after P120 (Figure 4O). Loss of UBE3A expression was properly achieved in striatum (Supplemental Figure 3, A and B) but had no significant effect on the excitability and synaptic transmission (Figure 4, O–U, and Supplemental Table 4). The F-I curves overlapped between all groups (Figure 4R). Additionally, the rheobase along with input resistance and sEPSC frequency were similar among all groups (Figure 4, Q–U, and Supplemental Table 4). These results indicate that loss of UBE3A after P45 has no effect on electrophysiological phenotypes.

### Lever press learning deficits are only partially rescued by P21 Ube3a gene reinstatement.

**Figure 5:**
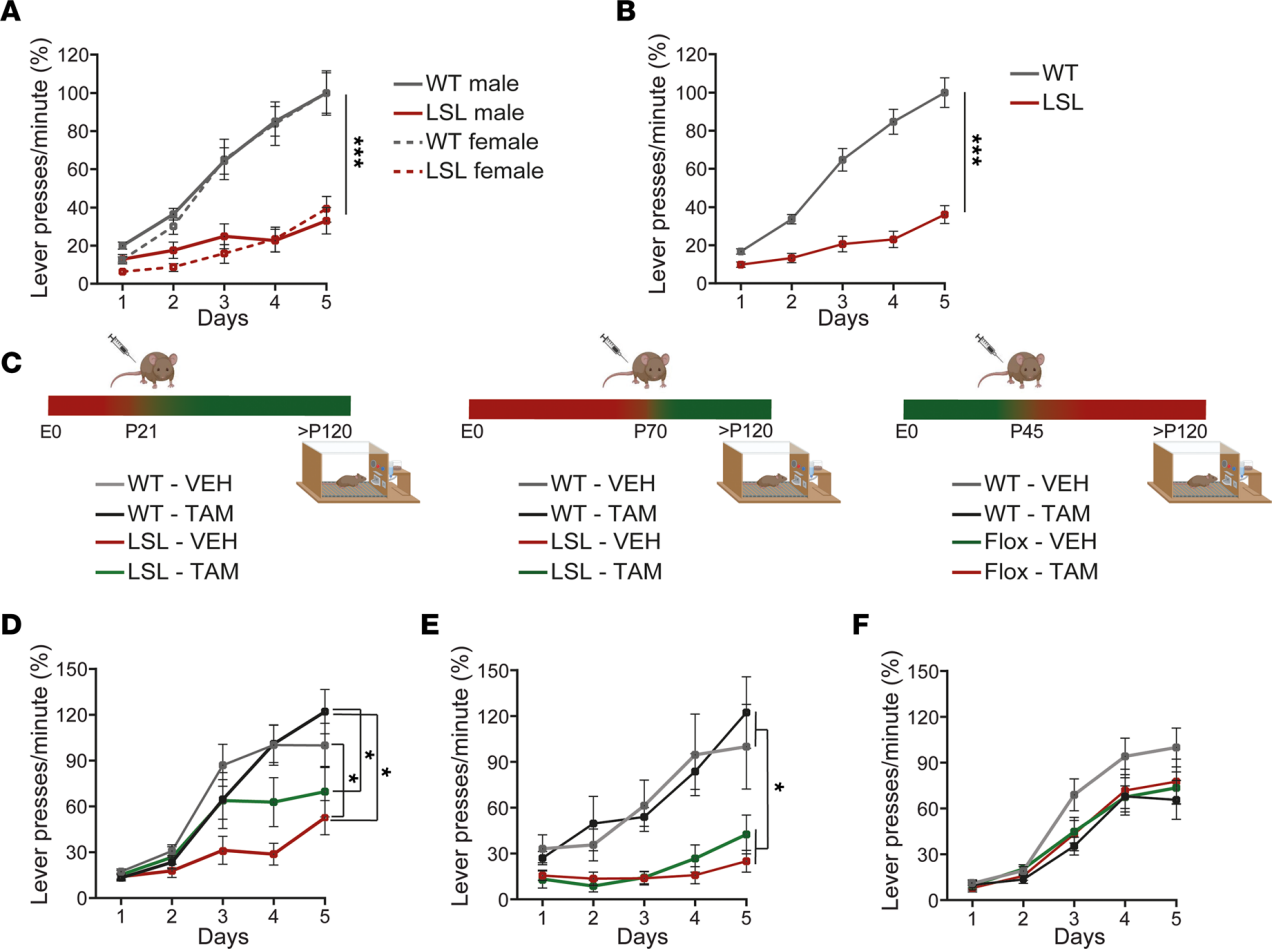
A critical window for rescuing lever press deficits. (**A** and **B**) Lever press rates during the fixed ration 1 (1 pellet rewarded for 1 lever press) phase, split on sexes (**A**), 2-way RM ANOVA, (*F_1, 50_* = 0.10, *P* = 0.74) and combined (**B**) 1-way RM ANOVA (*F_1, 50_* = 43.93, *P* < 0.001). (**C**) Schematic representation of *Ube3a* gene reinstatement (left, middle) or deletion (right) at indicated postnatal days and the time point of the lever press experiments. (**D**–**F**) Lever press rates, 2-way RM ANOVA: (**D**) (*F_1,_* *_34_* = 1.44, *P* = 0.24), with genotype effect (*F_1,_* *_34_* = 8.13, *P* = 0.007); post hoc Bonferroni’s: LSL-VEH against WT-VEH (*P* = 0.03), LSL-VEH against WT-TAM (*P* = 0.03), LSL-VEH against LSL-TAM (*P* = 0.6), LSL-TAM against WT-TAM (*P* = 1); (**E**) (*F_1,_* *_40_* = 0.008, *P* = 1), with genotype effect (*F_1,_* *_40_* = 19, *P* = 0.0001); post hoc Bonferroni’s: LSL-VEH against WT-VEH (*P* = 0.02), LSL-VEH against WT-TAM (*P* = 0.01), LSL-VEH against LSL-TAM (*P* = 1), LSL-TAM against WT-TAM (*P* = 0.03); (**F**) (*F_1,__54_* = 2.23, *P* = 0.14), and no effect of the genotype (*F_1,_* *_54_* = 0.63, *P* = 0.43) or treatment (*F_1,_* *_54_* = 2.23, *P* = 0.143). Sample size (*N* = mice): in (**A**) WT males: *N* = 14 and females: *N* = 11, LSL males: *N* = 14 and females: *N* = 13, (**B**) LSL: *N* = 27, WT: *N* = 25, (**D**) WT-VEH: *N* = 10; WT-TAM: *N* = 10; LSL-VEH, *N* = 9; LSL-TAM: *N* = 10) (**E**) WT-VEH: *N* = 12; WT-TAM: *N* = 12; LSL-VEH, *N* = 10; LSL-TAM: *N* = 10); (**F**) WT-VEH: *N* = 16; WT-TAM: *N* = 14; Flox-VEH, *N* = 13; Flox-TAM: *N* = 15). Data represent mean ± SEM. *P ≤ 0.05, ***P ≤ 0.001.

The lever press task is a commonly used behavioral readout in rodents to investigate striatal function (50), and previous data showed that male AS mouse models have impaired initial learning in this task (27). We assessed the presence of a lever press learning phenotype in LSL and WT male and female mice. For this task, the mice learn to associate 1 lever press with a sugar pellet over a period of 5 consecutive days. An increased number of lever presses reflects better learning. Both male and female LSL mice performed significantly fewer lever presses (Figure 5, A and B), without any sex differences. The learning curve in the LSL mice was significantly lower than WT littermate mice.

Next, we investigated the critical window to rescue the lever press learning deficits. We hypothesized that the electrophysiological critical window overlaps with the window for the lever press learning. To test this hypothesis, we reinstated the *Ube3a* gene either at P21 or P70 by injecting the LSL-Cre mice either with TAM or vehicle at these 2 time points (Figure 5C). Upon early, P21, UBE3A reinstatement, the LSL-TAM mouse learning curve only partially returned to the WT-TAM and WT-VEH levels (Figure 5D). Factorial ANOVA showed no significant interaction between genotype (WT, LSL) and treatment (VEH, TAM) but showed a significant effect of genotype. Post hoc analysis revealed that LSL-VEH mice were significantly different from WT-VEH and WT-TAM, but not LSL-TAM, while LSL-TAM were not different from any groups (Figure 5D). Thus, P21 reinstatement led to a partial rescue of the learning deficits in the lever press task. However, with late reinstatement of UBE3A expression, at P70, the learning deficits in LSL-TAM mice were not rescued. The learning curve in LSL-TAM and LSL-VEH overlapped and was lower than WT-TAM and WT-VEH (Figure 5E). Factorial ANOVA showed no significant interaction between genotype (WT, LSL) and treatment (VEH, TAM) but showed a significant effect of genotype. Post hoc analysis revealed that LSL-VEH and LSL-TAM were not significantly different from each other but were different from WT-VEH and WT-TAM (Figure 5E).

To investigate if UBE3A is needed after P45 for the lever press learning task after brain development has taken place, we made use of the Flox-Cre mice in which the *Ube3a* gene is deleted upon TAM injection. Flox-Cre mice were injected with TAM or VEH at P45 and tested on the lever press test 11 weeks later (Figure 5C). Although the learning curve for WT-VEH was on average higher than the rest of the groups, we found that loss of UBE3A after P45 had no significant effect on the lever press learning.

## Discussion

Here we used several *Ube3a* mouse models to identify striatal specific phenotypes, determine their developmental profile, and identify their critical windows for reversibility. We showed that absence of UBE3A results in impaired striatal development characterized by increased excitability, higher input resistance, and lower excitatory transmission onto MSNs. The electrophysiological deficits emerge at P21, and their magnitude increases during further development. *Ube3a* gene reinstatement at P21 fully rescues the increase in firing rates and input resistance but only partially rescues the excitatory synaptic transmission. These electrophysiological deficits are not rescued by P70 gene reinstatement, while they remain unaffected if deletion of *Ube3a* gene is induced after normal brain development at P45. Additionally, we showed that the initial learning in the lever press task is only partially rescued by P21 reinstatement but unaffected if the gene is deleted after normal brain development. Our electrophysiological findings are in line with other studies showing the maturation profile in rodent striatum. Moreover, we validated previously observed changes in striatal function as assessed by the lever press task. We further identified the critical therapeutic window in which treatment has to be initiated to prevent striatal dysfunction.

Adult *Ube3a* gene reinstatement in striatum resulted in approximately 62% of protein level compared with WT. This is lower than what we obtained previously in the hippocampus and prefrontal cortex, where we observed a full reversal of electrophysiological phenotypes (19, 37). Although this could indicate that UBE3A levels were not high enough to get a full rescue, we believe that the inability to reverse striatal deficits in adult mice is reflecting the limited reversal of striatal deficits after the critical period of striatal development has taken place, rather than a lack of sufficient UBE3A levels at the time of testing. This is strongly supported by the notion that deletion of *Ube3*a in adult mice (resulting in <10% of UBE3A) does not affect striatal function. Moreover, mice exclusively expressing UBE3A-Iso3 at 70% of total UBE3A levels also show no behavioral deficits (36).

It is well established that neuronal excitability and synaptic transmission are affected by loss of UBE3A in different brain areas (reviewed by Rotaru et al., ref. 51), but few studies investigated when these deficits emerge. One study used whole-cell patch clamp to investigate the excitatory synaptic events in the visual cortex of AS mice at 3 time points during development: just before eye opening (P10), juvenile (P25), and adult (P100) (32). Similar to our findings, the authors found that at P10, the frequency of the EPSCs was indistinguishable between WT and AS mice, while after P25 the frequency failed to develop normally and remained low for AS mice. A second study used intrinsic signal optical imaging at P20, P40, and P85, to investigate the development of the visually evoked neuronal activity from the primary visual cortex to higher order visual areas in AS and WT controls, and showed that AS mice fail to develop normal responses (52). Here, we provided an extended developmental profile of the striatal neurons and identified a specific time window, between P15 and P21, when UBE3A protein is crucial for striatal maturation.

Our previous data showed that the electrophysiological phenotypes of neurons in the PFC and hippocampus from AS mice could be restored by adult reinstatement of *Ube3a* (19, 37). If these results can be extrapolated to other cortical areas, this could indicate that the failure to rescue motor deficits in adult mice is not caused by a failure to correct neurons in the motor cortex. In contrast, the DMS, a subcortical area, is strongly affected by absence of UBE3A during early postnatal neurodevelopment, and failed to restore its function upon *Ube3a* gene reinstatement in adult mice. This suggests that these deficits of the DMS are potentially underlying some of previously described motor phenotypes in AS mice, such as rotarod learning (19) as well as operant conditioning (27), and possibly dictate the critical period for rescue of these AS mouse behaviors.

A scientific limitation of our study is the use of brain-wide mutants, rather than mutants in which *Ube3a* is specifically deleted in MSNs from the DMS only. Thus, the precise contribution of the specific brain areas to the behavioral and electrophysiological phenotypes we described remains to be shown. A translational limitation of our study is that it remains to be determined how the developmental window for striatal maturation, as assessed in this study, aligns with striatal development of individuals with AS.

### Striatal function in individuals with AS.

The striatum is involved in motor learning, speech, emotional regulation, and cognitive function (53–60), phenotypes that are all negatively affected in individuals with AS (13). Therefore, it is conceivable that changes in this brain area may underlie at least some of the major developmental deficits observed in patients with AS, an idea already proposed by several studies (25–27). The data we show here further support this hypothesis. First, one of the major deficits in patients with AS is profound motor dysfunction (61). The electrical activities of MSNs correlate with movement (34, 62, 63), and pathology within different BG circuits leads to movement disorders (12, 64, 65). AS mice also show readily observable motor deficits (51), and our previous data showed that such motor deficits are not arising from cerebellar deficits (21). Second, individuals with AS have a lack of speech, and the striatum has been shown to be important for speech (66). Third, repetitive and stereotyped movements and other behavioral abnormalities are a prominent feature of AS and commonly observed in ASDs in which the striatum is strongly involved (8, 67–70).

### Striatal neurodevelopment.

In mice, striatal maturation takes place within the first 4 weeks postnatally and includes a decrease in input resistance of MSNs, leading to a lower excitability, increased soma size, and increased spine density (44, 45). Our data from WT littermates are in line with such previous reports. We showed a gradual increase in MSNs’ excitability as the F-I curves showed a right shift with age, accompanied by decreased input resistance, likely due to an increase over age in the expression of inwardly rectifying potassium channels (primarily Kir2.1 and Kir2.3) (41, 71, 72) and leak potassium channels (KCNK channels) (73). We also observed an increased capacitance with age, likely reflecting an increase in neuronal size (74). Changes in synaptic transmission included an increase in sEPSCs, most likely resulting from an increase in the number of excitatory synapses (45, 72). In LSL mice, all of these parameters showed a strong delay and remained at an immature state.

Perturbing striatal activity as early as P7 has been shown to alter the MSNs’ properties, leading to significant changes in circuit maturation (38, 75, 76). Our data showed that the absence of UBE3A caused the emergence of electrophysiological phenotypes around P21, including changes in both excitability and excitatory synaptic transmission in the DMS. Nevertheless, already at P15 the speed of development was decreased in LSL mice, suggesting that these electrophysiological phenotypes are most likely preceded by molecular changes dependent on UBE3A, which remain to be identified. Our data point to a lack of upregulation of inwardly rectifying potassium channels (primarily Kir2) (41, 71, 72) and leak potassium channels (KCNK channels) (73), which likely translates into increased input resistance and thus increased excitability (72). During postnatal development of the BG, the motor programs start to manifest, leading to activity-dependent synapse formation in the striatum (77–80), a process that involves specific patterns of gene activity (43). Around P21 we also observed a lower frequency of sEPSCs in LSL mice, which may reflect a lack of synapse formation onto MSNs (81). MSNs from the DMS receive inputs from both PFC layer 5 pyramidal neurons and the thalamus (82). A recent study proposed that in AS mice only the thalamic inputs onto MSNs are decreased (83). These data are in line with functional MRI studies in patients with AS, which point to a decreased thalamo-striatal functional connectivity (26).

In line with striatal dysfunction in AS mice, we also validate previously published findings that AS mouse models show robust deficits in operant conditioning such as the lever press task (27). Another motor task, strongly dependent on striatum, is the rotarod learning, which we also have shown previously to be impaired in LSL mice (19). Using our inducible *LSL-Cre* (19, 37) mouse model, we now show that there is no reversal of the electrophysiological phenotypes when *Ube3a* is reinstated at P70, while the P21 reinstatement restores the excitability entirely and the synaptic transmission partially. This period for the rescue of the excitability in the MSNs aligns with the critical period for the rotarod behavioral tests, which is also limited to the first 21 days postnatally (19). However, the lever press task could only be reversed partially by P21 *Ube3a* gene reinstatement, suggesting that other electrophysiological changes, including excitatory synaptic transmission along with other changes not identified here, may be responsible for this behavioral task.

### The cause of striatal electrophysiological phenotypes.

The observed striatal electrophysiological phenotypes may arise exclusively from the loss of UBE3A in MSNs, being thus independent of the functional changes observed in other areas of the brain (51). However, this scenario is very unlikely, since MSN maturation is (also) driven by its inputs. The initial establishment of the cortico-striatal circuitry is a prerequisite for the proper functioning of BG circuits and the behavior they control (62, 84). In a *Shank3B^–/–^* mice, it was shown that an increased excitatory drive from cortex into the striatum leads to faster maturation of MSNs with consequences in adulthood, and rescuing the cortical hyperexcitability was sufficient to rescue the striatal maturation deficits (38). We showed previously that in AS mice, the PFC layer 5 pyramidal neurons, which have outputs directly onto MSNs from the DMS, have an increased excitation-to-inhibition ratio (36, 37), potentially affecting the MSNs’ speed of maturation. Yet, our data point to a stalled MSN development around P21, indicating a distinctly different mechanism causing striatal deficits compared with *Shank3B^–/–^* mice. A second factor contributing to the maturation of the BG circuits are the local inhibitory connections between MSNs (75). We show that MSNs are hyperexcitable at P21 in LSL mice, which may increase the inhibitory transmission between themselves, leading to further changes to their own wiring. A third crucial factor for striatal maturation is dopaminergic transmission. Dopamine, via the PKA pathway, has significant effects on spinogenesis, the major site for excitatory synaptic transmission, during the second week postnatal (76). We and others have identified changes in dopaminergic transmission in different areas of the striatum (33, 85–87). Moreover, recently, it was proposed that dopamine acts as a critical period signal in striatal maturation (72).

The striatal deficits observed by us and others (27) in the mature striatum of AS mice, may thus be the result of deficits at any of the 3 levels explained above or a combination of them. To identify the critical brain regions involved in shaping striatal function as well as the related behavioral changes, future experiments should focus on deleting *Ube3a* during early (prenatal) brain development in cortical, striatal, or dopaminergic neurons. Our study indicates that combining these approaches with electrophysiological measurements, as well as behavioral testing (motor function, anxiety measures, and repetitive behavior), will allow us to further understand how neuronal changes lead to behavioral dysfunction. Such knowledge is critical to identify therapies and to understand when during development these therapies should be administered to get optimal therapeutic benefit.

## Methods

### Experimental model details

For this study, we used 2 mutant *Ube3a* mouse lines: *Ube3a^mStop/p+^* (*Ube3a^tm1Yelg^*; MGI:5704099) (19) and *Ube3a^mFlox/p+^* (*Ube3a^tm1.1Bdph^*; MGI:5882092) (48, 49). The *Ube3a^mFlox/p+^* (*Ube3a^tm1.1Bdph^*; MGI:5882092) mouse line was provided by the Philpot laboratory, the Department of Cell Biology & Physiology, University of North Carolina at Chapel Hill (Chapel Hill, North Carolina, USA). The *Ube3a^tm1Yelg^* line was maintained in the 129S2 background (129S2/SvPasCrl) by crossing male *Ube3a^m+/pStop^* mice with female 129S2 WT mice. The *Ube3a^tm1.1Bdph^* line was maintained in the C57BL/6J background (Charles River) by crossing male *Ube3a^m+/pFlox^* mice with C57BL/6J WT females. The mouse lines do not show audiogenic seizures (35). Depending on the experiment (see below and in the results), mice were between the ages of P15 and P180. For all the experiments, we used fairly equal percentages of male and female mice in the F1 hybrid 129S2 C57BL/6J background, as explained below. The experimental unit represented neurons for electrophysiological recordings and animals for the behavioral experiments. Genotypes were blinded during the experiment and the analysis of the data.

To identify the electrophysiological phenotype and its neurodevelopmental profile (Figures 1–3 and Figure 4, A–N) and to test the lever press learning (Figure 5, A and B), we crossed female 129S2*Ube3a^m+/pStop^* mice with WT C57BL/6J males (Charles River) to generate *Ube3a^mStop/p+^* (referred to as LSL) and their WT littermate controls, *Ube3a^m+/p+^* (referred to as WT), in a 129B6F1 hybrid background. The mice in this group were between 15 and 180 days old, as further detailed in the results. To establish the critical window of the described phenotypes (Figure 4, A–N, and Figure 5, C–E) we crossed female 129S2*Ube3a^m+/pStop^* with homozygous B6Tg(CAG-Cre/Esr1*)5Amc/J (MGI:2182767; The Jackson Laboratory) (88), to generate Ube3a^mStop/p+^-Cre^Esr1*+^ mice, whereupon TAM treatment at any time point, UBE3A levels are reliably restored, as previously shown (19, 37), and their WT littermate controls Ube3a^m+/p+^-Cre^Esr1*+^, in a 129B6F1 hybrid background. Both groups were treated with either TAM or oil (hereby referred to as LSL-TAM, LSL-VEH, WT-TAM, and WT-VEH, respectively). To test if UBE3A is necessary after neurodevelopment (Figure 4, O–U, and Figure 5, C and F), we crossed female B6*Ube3a^m+/pFlox^* mice with homozygous 129S2Tg(CAG-CRE/Esr1*)5Amc/J (MGI:2182767) male mice to generate Ube3a^mFlox/p+^-Cre^Esr1*+^, whereupon TAM treatment, UBE3A levels are reliably decreased to the levels expressed by the *Ube3a^mStop/p+^* mice (48, 49) and their WT littermate controls Ube3a^m+/p+^-Cre^Esr1*+^ in a 129B6F1 hybrid background. Both groups were treated with either TAM or oil (referred to as Flox-TAM and Flox-VEH, WT-TAM, or WT-VEH, respectively). For simplicity, we used an abbreviated nomenclature for the different genotypes and treatments used in this study (Table 2). The mice used in these experiments were between P70 and P180 as detailed in the results.

TAM (0.10 mg of TAM/g of body weight) or vehicle (oil) was delivered by intraperitoneal injections for 5 consecutive days as previously described (19, 37, 48, 49). TAM was diluted in sunflower oil at a concentration of 20 mg/mL.

### Electrophysiology

#### Slices.

Electrophysiological experiments were performed on animals between P15 and P180. Coronal striatal slices of 300 μm were prepared in ice-cold, modified ACSF containing the following (in mM): 125 NaCl, 3 KCl, 1.25 NaH_2_PO_4_, 26 NaHCO_3_, 10 glucose, 7 MgSO_4_, and 0.5 CaCl_2_, as previously described (37). The recording chamber was maintained at 28°C–30°C when superfused at 2 mL/min with normal ACSF containing the following (in mM): 125 NaCl, 3 KCl, 1.25 NaH_2_PO_4_, 26 NaHCO_3_, 10 glucose, 1 MgSO_4_, and 2 CaCl_2_, pH 7.3–7.4, when bubbled with 95% O_2_/5% CO_2_. Cells were visualized using a Nikon microscope (ECLIPSE E600FN) with infrared illumination and differential interference contrast video microscopy. Patch electrodes (3–4 MΩ) were backfilled with internal solution that contained the following (in mM): K-gluconate 125, NaCl 10, HEPES 10, EGTA 0.2, MgATP 4.5, NaGTP 0.3, and K-phosphocreatine 10, adjusted for pH to 7.2–7.4 using KOH. Whole-cell recordings were obtained from MSNs in the DMS using Multiclamp 700B amplifiers (Axon Instruments). Signals were low-pass–filtered at 4 kHz and digitized at 20 kHz using Digidata 1440A (Molecular Devices) acquisition interfaces, and acquisition and analysis were performed using Clampex (Axon Instruments) or Minianalysis software (Synaptosoft).

#### Current clamp.

For current clamp recording, series resistance and pipette capacitance were monitored and cancelled using bridge and capacitance neutralization. To obtain the passive properties and the firing pattern, we recorded voltage responses in current clamp mode from neurons held at around –80 mV, while we injected a family of 500 ms square pulses starting from –100 pA with 10 pA increments, delivered at 0.2 Hz.

#### Voltage clamp.

The pipette capacitance was compensated, and series resistance was continuously monitored but was not compensated. Only recordings with a stable series resistance of less than 20 MΩ were used for analysis. sEPSCs were recorded in gap-free protocol in Clampex, for 10 minutes, while the cells were voltage clamped at –70 mV.

### Western blot

For the Western blot analysis, frontal cortex and striatal tissue were dissected from adult mice and immediately frozen in liquid nitrogen. The lysates were prepared by adding lysis buffer (10 mm Tris-HCl, pH 6.8, 2.5% SDS) supplemented with protease inhibitor mixture (P8340, MilliporeSigma, 1:100 dilution) to the tissue, and homogenization was achieved by sonication. After centrifugation (3,000*g*, at 4°C for 5 minutes), supernatants were collected, and concentration was measured using the BCA protein assay kit (Thermo Fisher Scientific). Lysate concentrations were adjusted to 1 mg/mL. A total of 20 μg of each sample was loaded on the gel, and a semidry transfer was performed (Trans-Blot Turbo Transfer System, Bio-Rad). The blotted nitrocellulose membrane was probed with antibodies directed against E6AP (MilliporeSigma, E8655, 1:1,000 dilution) and actin (MilliporeSigma, MAB1501R, 1:20,000 dilution). A fluorophore-conjugated secondary goat anti-mouse antibody (Westburg, IRDye 800CW, 926-32210, 1:15,000) was used, and the protein was detected using Odyssey Scanner system (LI-COR Biosciences). Quantification was done using Odyssey 3.0 software (LI-COR Biosciences).

### Instrumental conditioning

For the instrumental conditioning, we followed the protocol described previously (89–91). We used Med Associates mouse operant chambers (Noldus) that contained a food magazine, with a Bio-Serv 14 mg pellet dispenser placed next to the lever. Mice were single caged and handled by the experimenter daily for 7 days before the experiment. Food restriction began 5 days before the experiment, allowing for a gradual weight loss of 15% from their ad libitum weight. On the day before the experiment, the mice received 2 sugar pellets in their cages. During the learning phase that lasted 5 days, mice had to press the lever once to receive 1 pellet.

### Statistics

#### Analysis of passive and active properties.

Capacitance was obtained at the beginning of each recording using the membrane test tool in Clampex where a 10 mV voltage test was delivered from –70 mV voltage holding. Input resistance was obtained offline, by fitting a linear regression on the voltage-current data points obtained in the passive domain of the MSNs. The rheobase was the level of the 500 ms current injection that generated at least 1 AP. The firing frequency was calculated by counting the number of APs at each 500 ms current injection step. To obtain the F-I relationship, we plotted the average firing frequency against the value of the current injection. The maximum firing frequency was the maximum number of APs each MSN produced with increasing depolarization before spikes dropped. The F-I slope was obtained for each MSN by fitting a linear regression on the firing frequency plotted against the injected current data points. The AP threshold was calculated at rheobase level as the voltage, where dV/dt = 30 mV/ms. To obtain the relationship between the membrane depolarization generated by positive current injection and the number of APs that followed, we calculated the voltage at 25 ms after the current injection and counted the number of APs triggered by that current injection.

#### EPSC data analysis.

EPSCs were detected using Mini analysis software (Synaptosoft). To analyze the frequency, events were counted over 10 minutes of recording. To obtain the average events, for each cell, at least 100 nonoverlapping events were detected and averaged. The peak amplitude of the average sEPSCs was measured relative to the baseline current.

#### Regression analysis.

To obtain the best fit line to each parameter measured at different postnatal time points, we used the regression analysis and curve estimation module in SPSS. The data from LSL obtained at different time points were pulled and a series of mathematical functions including linear, logarithmic, inverse, quadratic, cubic, power, compound, S-curve, logistic, growth, and exponential were compared based on their relative goodness of fit. The single dependent variable (measured parameter) was predicted by a single independent variable (postnatal day) (Supplemental Table 3). After identifying the best curve for each parameter, we obtained the genotype-specific fit for each parameter (Table 1).

#### General statistics.

The statistical analysis was performed in IBM SPSS software. The statistical tests performed for data are indicated in figure legends. The exact *P* values for each statistical test are reported either in figure legends or in tables, as indicated. *P* ≤ 0.05 was considered statistically significant. When only 2 groups were compared, we used the 2-tailed, unpaired *t* test. When more than 2 groups were compared, we used either 1-way RM ANOVA, 2-way/factorial ANOVA, or 2-way RM ANOVA. If a significant effect of the independent factors was observed, we applied post hoc analysis, as indicated.

Specifically, to test the effect of genotype (WT, LSL) on the electrophysiological properties, we used the following: 1-way RM ANOVA (firing frequency as repeated measures) in Figure 1B, Supplemental Figure 1B, and Figure 2B and the 2-tailed unpaired *t* test in Figure 1, C–E, G, H, J, M, and N, and Supplemental Figure 1, E and F. To test the interaction between the genotypes and age and of both factors on the electrophysiological phenotypes, we used factorial ANOVA with genotype (WT, LSL) and age (P15, P21, P35, P45, P130) as independent variables followed by post hoc least significant differences test (Supplemental Tables 1 and 2), in Figure 2, C–F and H, and Supplemental Figure 2A.

To analyze the interaction between treatment and genotypes and of both factors on the electrophysiological phenotypes and the *Ube3a* gene reinstatement/deletion, we used the following: 2-way RM ANOVA (firing frequency as RM) followed by post hoc Bonferroni’s in Figure 4, D, E, and R (independent variable treatment: VEH, TAM), and genotype (WT, LSL, in Figure 4, D and E; or WT, Flox, in Figure 4R), and 2-way ANOVA followed by post hoc Bonferroni’s (Supplemental Table 4) in Figure 4, E–G, L–N, and S–U; and Supplemental Figure 3, B–D.

To test the interaction between sex (male, female) and genotype (WT, LSL) and of both factors on the lever press phenotype, we used the following: 2-way RM ANOVA (days as repeated measures) in Figure 5A and 1-way RM ANOVA in Figure 5B. To test the interaction of treatment with genotypes and of both factors on the lever press phenotypes, we used 2-way RM ANOVA (days as RM) followed by post hoc Bonferroni’s in Figure 4, D–F (independent variable treatment: VEH, TAM), and genotype (WT, LSL, in Figure 4, D and E; or WT, Flox, in Figure 4F).

### Study approval

All animal experiments were conducted in accordance with the European Commission Council Directive 2010/63/EU (CCD approval AVD101002016791, the Hague, the Netherlands). Mice were housed in individual ventilated cages and kept on a (normal) 12-hour light/12-hour dark cycle, at 22°C ± 2°C and had ad libitum access to food and water. Animal welfare was checked daily by animal caretakers and experimenters throughout the experiments. All cages contained basic bedding and nesting material.

## Author contributions

DCR designed, performed, and analyzed the electrophysiological experiments and wrote the manuscript. IW performed the Western blots and the behavioral experiments and contributed to the figures. MDV performed and analyzed electrophysiological and behavioral experiment and contributed to the manuscript. JVDB performed the behavioral experiments. YE designed and supervised the research and wrote the manuscript.

## Supplementary Material

Supplemental data

## Figures and Tables

**Table 1:**
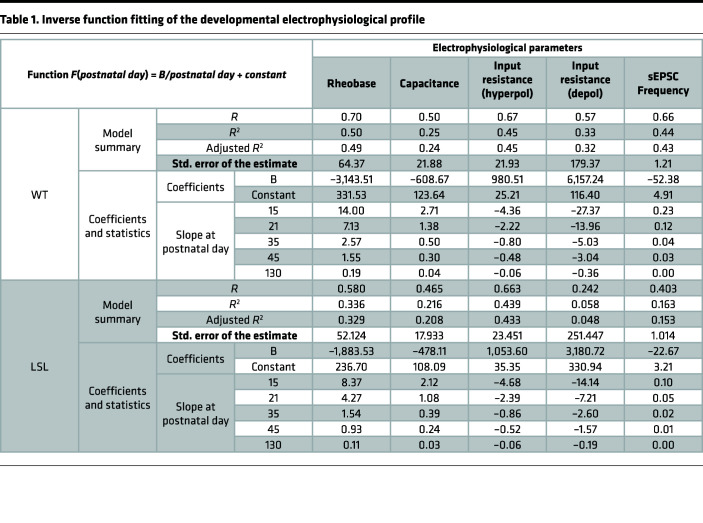
Inverse function fitting of the developmental electrophysiological profile

**Table 2:**
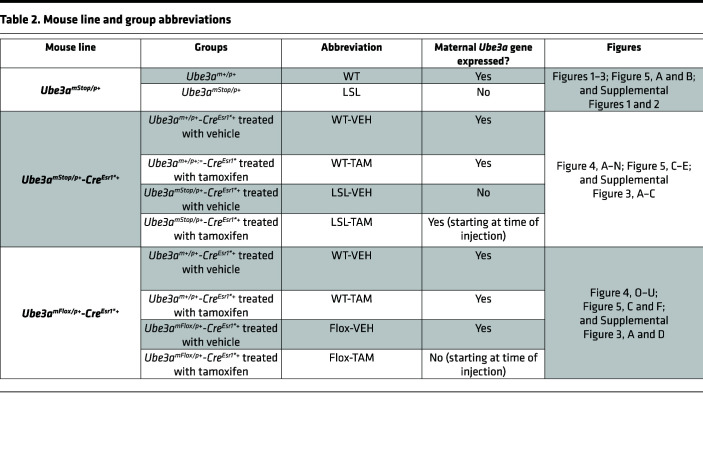
Mouse line and group abbreviations

## Version 1 02/22/2023

Electronic publication

## References

[1] Servetti M (2021). Neurodevelopmental disorders in patients with complex phenotypes and potential complex genetic basis involving non-coding genes, and double CNVs. Front Genet.

[2] Coe BP (2019). Neurodevelopmental disease genes implicated by de novo mutation and copy number variation morbidity. Nat Genet.

[3] Thapar A (2017). Neurodevelopmental disorders. Lancet Psychiatry.

[4] Hensch TK (2004). Critical period regulation. Annu Rev Neurosci.

[5] Takesian AE, Hensch TK (2013). Balancing plasticity/stability across brain development. Prog Brain Res.

[6] Niwa M (2010). Knockdown of DISC1 by in utero gene transfer disturbs postnatal dopaminergic maturation in the frontal cortex and leads to adult behavioral deficits. Neuron.

[7] Peng Y (2016). The autism-associated MET receptor tyrosine kinase engages early neuronal growth mechanism and controls glutamatergic circuits development in the forebrain. Mol Psychiatry.

[8] Di Martino A (2011). Aberrant striatal functional connectivity in children with autism. Biol Psychiatry.

[9] Forcelli PA (2012). Neonatal exposure to antiepileptic drugs disrupts striatal synaptic development. Ann Neurol.

[10] Biane JS (2015). Motor cortex maturation is associated with reductions in recurrent connectivity among functional subpopulations and increases in intrinsic excitability. J Neurosci.

[11] Sebastianutto I (2017). Alterations of striatal indirect pathway neurons precede motor deficits in two mouse models of Huntington’s disease. Neurobiol Dis.

[12] Yokoi F (2011). Motor deficits and decreased striatal dopamine receptor 2 binding activity in the striatum-specific Dyt1 conditional knockout mice. PLoS One.

[13] Williams CA (2010). The behavioral phenotype of the Angelman syndrome. Am J Med Genet C Semin Med Genet.

[14] Bindels-de Heus GCB (2020). An overview of health issues and development in a large clinical cohort of children with Angelman syndrome. Am J Med Genet A.

[15] Sadhwani A (2021). Developmental skills of individuals with Angelman syndrome assessed using the Bayley-III. J Autism Dev Disord.

[16] Hensch TK (2005). Critical period plasticity in local cortical circuits. Nat Rev Neurosci.

[17] Meng L (2015). Towards a therapy for Angelman syndrome by targeting a long non-coding RNA. Nature.

[18] Milazzo C (2021). Antisense oligonucleotide treatment rescues UBE3A expression and multiple phenotypes of an Angelman syndrome mouse model. JCI Insight.

[19] Silva-Santos S (2015). Ube3a reinstatement identifies distinct developmental windows in a murine Angelman syndrome model. J Clin Invest.

[20] Angelman H (1965). ‘Puppet’ children a report on three cases. Dev Med Child Neurol.

[21] Bruinsma CF (2015). Dissociation of locomotor and cerebellar deficits in a murine Angelman syndrome model. J Clin Invest.

[22] Shepherd GMG (2013). Corticostriatal connectivity and its role in disease. Nat Rev Neurosci.

[23] Alexander GE, Crutcher MD (1990). Functional architecture of basal ganglia circuits: neural substrates of parallel processing. Trends Neurosci.

[24] Griffiths KR (2014). Translational studies of goal-directed action as a framework for classifying deficits across psychiatric disorders. Front Syst Neurosci.

[25] Aghakhanyan G (2016). From cortical and subcortical grey matter abnormalities to neurobehavioral phenotype of angelman syndrome: a voxel-based morphometry study. PLoS One.

[26] Yoon HM (2020). Disrupted functional and structural connectivity in Angelman syndrome. AJNR Am J Neuroradiol.

[27] Hayrapetyan V (2014). Region-specific impairments in striatal synaptic transmission and impaired instrumental learning in a mouse model of Angelman syndrome. Eur J Neurosci.

[28] Peak J (2019). From learning to action: the integration of dorsal striatal input and output pathways in instrumental conditioning. Eur J Neurosci.

[29] Yin HH, Knowlton BJ (2006). The role of the basal ganglia in habit formation. Nat Rev Neurosci.

[30] Balleine BW, O’Doherty JP (2010). Human and rodent homologies in action control: corticostriatal determinants of goal-directed and habitual action. Neuropsychopharmacology.

[31] Gruber AJ, McDonald RJ (2012). Context, emotion, and the strategic pursuit of goals: interactions among multiple brain systems controlling motivated behavior. Front Behav Neurosci.

[32] Yashiro K (2009). Ube3a is required for experience-dependent maturation of the neocortex. Nat Neurosci.

[33] Berrios J (2016). Loss of UBE3A from TH-expressing neurons suppresses GABA co-release and enhances VTA-NAc optical self-stimulation. Nat Commun.

[34] Kupferschmidt DA (2017). Parallel, but dissociable, processing in discrete corticostriatal inputs encodes skill learning. Neuron.

[35] Sonzogni M (2018). A behavioral test battery for mouse models of Angelman syndrome: a powerful tool for testing drugs and novel *Ube3a* mutants. Mol Autism.

[36] Avagliano Trezza R (2019). Loss of nuclear UBE3A causes electrophysiological and behavioral deficits in mice and is associated with Angelman syndrome. Nat Neurosci.

[37] Rotaru DC (2018). Adult Ube3a gene reinstatement restores the electrophysiological deficits of prefrontal cortex layer 5 neurons in a mouse model of Angelman syndrome. J Neurosci.

[38] Peixoto RT (2016). Early hyperactivity and precocious maturation of corticostriatal circuits in Shank3B(−/−) mice. Nat Neurosci.

[39] Gertler TS (2008). Dichotomous anatomical properties of adult striatal medium spiny neurons. J Neurosci.

[40] Kreitzer AC (2009). Physiology and pharmacology of striatal neurons. Annu Rev Neurosci.

[41] Shen W (2008). Dichotomous dopaminergic control of striatal synaptic plasticity. Science.

[42] Auger C, Marty A (2000). Quantal currents at single-site central synapses. J Physiol.

[43] Novak G (2013). Striatal development involves a switch in gene expression networks, followed by a myelination event: implications for neuropsychiatric disease. Synapse.

[44] Tepper JM (1998). Postnatal development of the rat neostriatum: electrophysiological, light- and electron-microscopic studies. Dev Neurosci.

[45] Sharpe NA, Tepper JM (1998). Postnatal development of excitatory synaptic input to the rat neostriatum: an electron microscopic study. Neuroscience.

[46] Thomas MSC (2009). Using developmental trajectories to understand developmental disorders. J Speech Lang Hear Res.

[47] Costa RM (2004). Differential corticostriatal plasticity during fast and slow motor skill learning in mice. Curr Biol.

[48] Sonzogni M (2019). Delayed loss of UBE3A reduces the expression of Angelman syndrome-associated phenotypes. Mol Autism.

[49] Judson MCCC (2016). GABAergic neuron-specific loss of Ube3a causes Angelman syndrome-like EEG abnormalities and enhances seizure susceptibility. Neuron.

[50] Yin HH (2004). Lesions of dorsolateral striatum preserve outcome expectancy but disrupt habit formation in instrumental learning. Eur J Neurosci.

[51] Rotaru DC (2020). Angelman syndrome: from mouse models to therapy. Neuroscience.

[52] Townsend LB (2020). Deficits in higher visual area representations in a mouse model of Angelman syndrome. J Neurodev Disord.

[53] Lehericy S (2005). Distinct basal ganglia territories are engaged in early and advanced motor sequence learning. Proc Natl Acad Sci U S A.

[54] Li J, Daw ND (2011). Signals in human striatum are appropriate for policy update rather than value prediction. J Neurosci.

[55] Liljeholm M (2012). Contributions of the striatum to learning, motivation, and performance: an associative account. Trends Cogn Sci.

[56] Wymbs NF (2012). Differential recruitment of the sensorimotor putamen and frontoparietal cortex during motor chunking in humans. Neuron.

[57] Diuk C (2013). Hierarchical learning induces two simultaneous, but separable, prediction errors in human basal ganglia. J Neurosci.

[58] Daniel R, Pollmann S (2014). A universal role of the ventral striatum in reward-based learning: evidence from human studies. Neurobiol Learn Mem.

[59] Gepshtein S (2014). Dopamine function and the efficiency of human movement. J Cogn Neurosci.

[60] Graybiel AM, Grafton ST (2015). The striatum: where skills and habits meet. Cold Spring Harb Perspect Biol.

[61] Williams CA (2006). Angelman syndrome 2005: updated consensus for diagnostic criteria. Am J Med Genet A.

[62] Plenz D, Kitai ST (1998). Up and down states in striatal medium spiny neurons simultaneously recorded with spontaneous activity in fast-spiking interneurons studied in cortex-striatum-substantia nigra organotypic cultures. J Neurosci.

[63] Murer MG (2002). Brain oscillations, medium spiny neurons, and dopamine. Cell Mol Neurobiol.

[64] Gittis AH, Kreitzer AC (2012). Striatal microcircuitry and movement disorders. Trends Neurosci.

[65] Yin HH (2017). The basal ganglia in action. Neuroscientist.

[66] Enard W (2009). A humanized version of Foxp2 affects cortico-basal ganglia circuits in mice. Cell.

[67] Dichter GS (2012). Reward circuitry dysfunction in psychiatric and neurodevelopmental disorders and genetic syndromes: animal models and clinical findings. J Neurodev Disord.

[68] Delmonte S (2013). Functional and structural connectivity of frontostriatal circuitry in autism spectrum disorder. Front Hum Neurosci.

[69] Langen M (2014). Changes in the development of striatum are involved in repetitive behavior in autism. Biol Psychiatry.

[70] Fuccillo MV (2016). Striatal circuits as a common node for autism pathophysiology. Front Neurosci.

[71] Cazorla M (2012). Striatal D2 receptors regulate dendritic morphology of medium spiny neurons via Kir2 channels. J Neurosci.

[72] Lieberman OJ (2018). Dopamine triggers the maturation of striatal spiny projection neuron excitability during a critical period. Neuron.

[73] Talley EM (2001). Cns distribution of members of the two-pore-domain (KCNK) potassium channel family. J Neurosci.

[74] Golowasch J (2009). Membrane capacitance measurements revisited: dependence of capacitance value on measurement method in nonisopotential neurons. J Neurophysiol.

[75] Kozorovitskiy Y (2012). Recurrent network activity drives striatal synaptogenesis. Nature.

[76] Kozorovitskiy Y (2015). Neuromodulation of excitatory synaptogenesis in striatal development. Elife.

[77] Hamasaki T (2003). Neuronal cell migration for the developmental formation of the mammalian striatum. Brain Res Brain Res Rev.

[78] Arlotta P (2008). Ctip2 controls the differentiation of medium spiny neurons and the establishment of the cellular architecture of the striatum. J Neurosci.

[79] Piontkewitz Y (2011). Abnormal trajectories of neurodevelopment and behavior following in utero insult in the rat. Biol Psychiatry.

[80] Sohur US (2014). Anatomic and molecular development of corticostriatal projection neurons in mice. Cereb Cortex.

[81] Rothwell PEE (2014). Autism-associated neuroligin-3 mutations commonly impair striatal circuits to boost repetitive behaviors. Cell.

[82] Ding J (2008). Corticostriatal and thalamostriatal synapses have distinctive properties. J Neurosci.

[83] Moreira-de-Sá A (2021). Motor deficits coupled to cerebellar and striatal alterations in Ube3a^m−/p+^ mice modelling Angelman syndrome are attenuated by adenosine A2A receptor blockade. Mol Neurobiol.

[84] Snyder-Keller AM (1991). Development of striatal compartmentalization following pre- or postnatal dopamine depletion. J Neurosci.

[85] Farook MF (2012). Altered serotonin, dopamine and norepinepherine levels in 15q duplication and Angelman syndrome mouse models. PLoS One.

[86] Riday TT (2012). Pathway-specific dopaminergic deficits in a mouse model of Angelman syndrome. J Clin Invest.

[87] Steinkellner T (2012). Ca(2+)/calmodulin-dependent protein kinase IIα (αCaMKII) controls the activity of the dopamine transporter: implications for Angelman syndrome. J Biol Chem.

[88] Hayashi S, McMahon AP (2002). Efficient recombination in diverse tissues by a tamoxifen-inducible form of Cre: a tool for temporally regulated gene activation/inactivation in the mouse. Dev Biol.

[89] Rossi MA (2012). Prefrontal cortical mechanisms underlying delayed alternation in mice. J Neurophysiol.

[90] Rossi MA, Yin HH (2012). Methods for studying habitual behavior in mice. Curr Protoc Neurosci.

[91] Yu C (2009). Genetic deletion of A2A adenosine receptors in the striatum selectively impairs habit formation. J Neurosci.

