# Supplementary material for: UBE3A expression during early postnatal brain development is required for proper dorsomedial striatal maturation
Source: JCI Insight. 2023 Feb 22;8(4):e166073. doi: 10.1172/jci.insight.166073 (PMC9977510; doi:10.1172/jci.insight.166073)
Supplement: Supplemental data [file jciinsight-8-166073-s086.pdf]

Supplementary material

Supplementary Figures

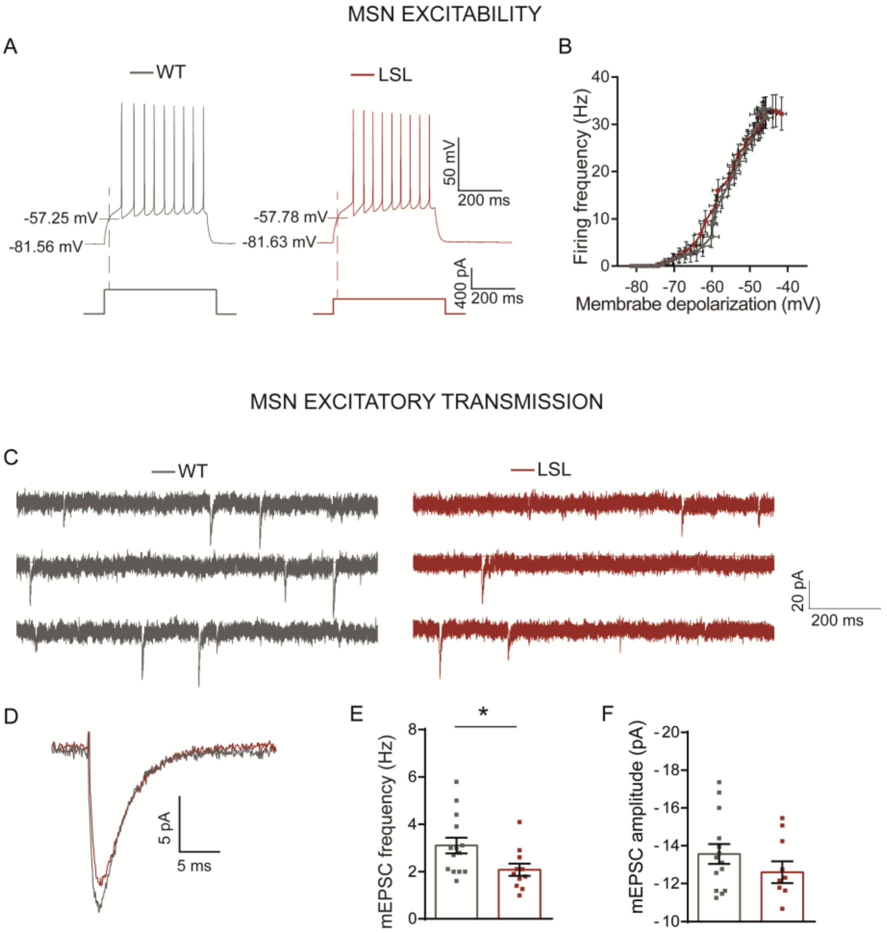

Supplementary Figure 1. Firing rates and miniature excitatory synaptic currents - *refers to Figure 1***.**

(A) Representative firing pattern of MSNs (top) obtained with current injection (bottom) showing that similar depolarization at 25ms (doted lines) after the current injection results in similar number of AP. **(B)** Firing frequency vs. membrane depolarization, one-way RM ANOVA ($F_{(1,31)}$ = 0.43, p = 0.52). **(C)** Representative recordings of mEPSCs. **(D)** Representative average mEPSCs. **(E)** mEPSCs frequency, two-tails unpaired t test (t = 2.30 df = 23, p = 0.03). **(F)** mEPSCs amplitude, two-tails unpaired t test (t = 1.37 df = 23, p = 0.18). Sample size (*N* = neurons/mice), for **B**: WT: *N* = 17/3; LSL: *N* = 16/3 and for **E, F**: WT: *N* = 14/7; LSL: *N* = 11/6. Data represents dot plots (one neuron) with mean ± SEM. $*p \leq 0.05$.

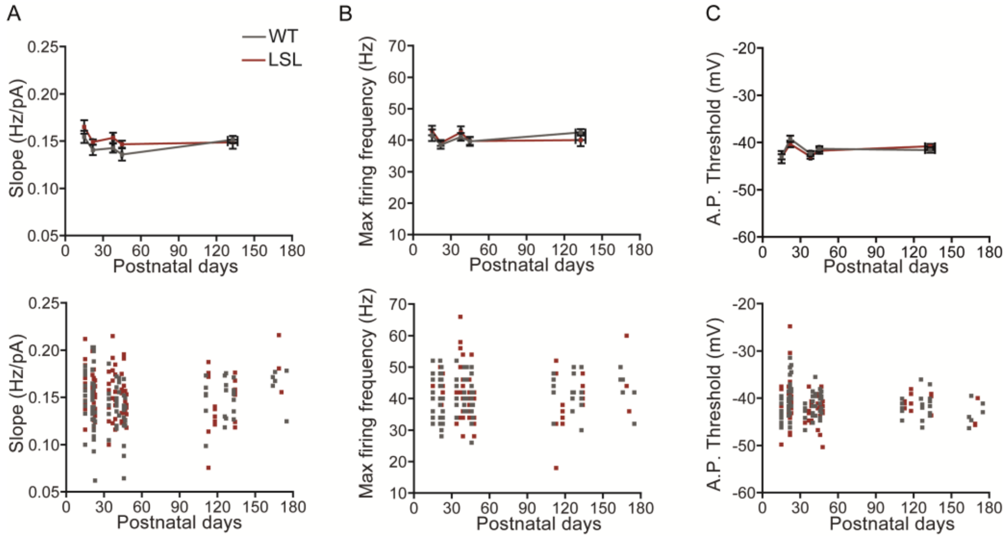

Supplementary Figure 2. Neurodevelopmental profile (Active properties) – *refers to Figure 2.*

(A) F-I Slope (top), mean ± SEM showing similar changes between LSL and WT over time (table S2); individual data obtained at different time points during development (bottom); regression analysis did not reveal a significant correlation for majority of the tested models (table 3); **(B)** Maximum firing rate mean ± SEM (top) showing similar changes for LSL and WT over time (table S2); individual data obtained at different time points during development (bottom); regression analysis did not reveal a significant correlation for majority of the tested model (table 3) **(C)** AP Threshold Mean ± SEM (top) showing similar changes between LSL and WT over time (table S2); individual data obtained at different time points during development 9bottom); regression analysis did not reveal a significant correlation for majority of the tested model (table 3).

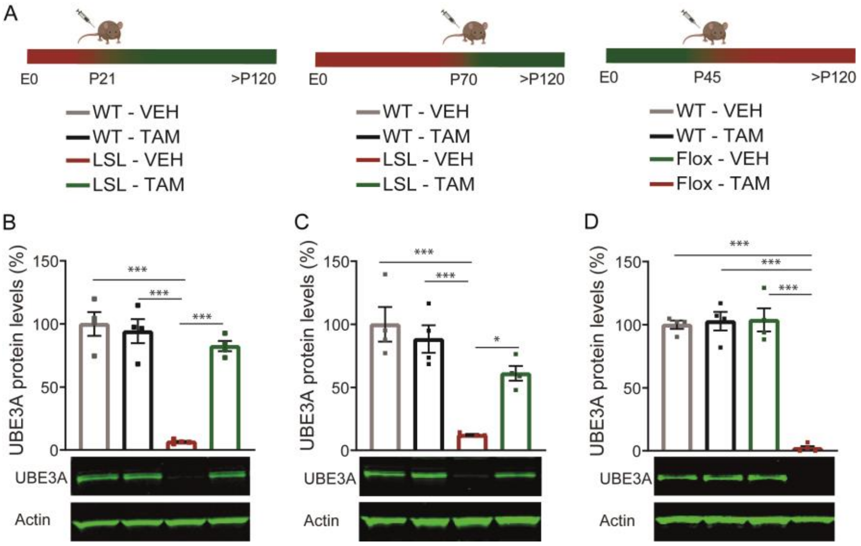

Supplementary Figure 3. UBE3A levels – *refers to Figure 4 and 5* **(A)** Schematic representation of *Ube3a* gene reinstatement (left, middle) or deletion (right) at different type points during development and the time point of Western blot quantification of UBE3A proteins **(B), (C), (D)** Relative UBE3A expression (top), Examples of Western blot analysis in striatum (bottom), after reinstatement of *Ube3* gene at P21 in (B), P70 in (C), and deletion of *Ube3* gene at P45 in (D). Two-way RM ANOVA: (B) ($F_{(1, 12)}$ = 33.66, p < 0.001), *Post hoc* Bonferroni: LSL-VEH against WT-VEH (p < 0.001), LSL-VEH against WT-TAM (p < 0.001), LSL-VEH against LSL-TAM (p < 0.001), LSL-TAM against WT-TAM (p = 1); (C) ($F_{(1, 12)}$ = 10.60, p = 0.007), *Post hoc* Bonferroni: LSL-VEH against WT-VEH (p < 0.001), LSL-VEH against WT-TAM (p < 0.001), LSL-VEH against LSL-TAM (p = 0.018), LSL-TAM against WT-TAM (p = 1); (D) ($F_{(1, 12)}$ = 72.27, p < 0.001), *Post hoc* Bonferroni: Flox-TAM against WT-VEH (p < 0.001), Flox-TAM against WT-TAM (p < 0.001), Flox-VEH against Flox-TAM (p < 0.001), Flox-VEH against WT-VEH (p = 1). Sample size: *N = 4* mice in each condition.: Data represents dot plots (one mouse) with mean ± SEM. ∗p ≤ 0.05, ∗∗∗p ≤ 0.001

Supplementary Tables

Supplementary Table 1. Developmental profile of the electrophysiological phenotypes (passive properties and synaptic transmission) – refers to Figure 1 and 2

Figure		*Parameter*	*Age*	*Genotypes (Mean ± SEM)*		*Statistics*	
				WT	*LSL*	*Test*	*p value*
Figure 2	*C*	*Rheobase (pA)*	*P15*	104.62 ± 9.25	100.77 ± 13.33	*Post hoc Anova, LSD test*	0.877
			P21	189.15 ± 9.87	150.79 ± 6.66		0.010
			P35	252.95 ± 19.33	180.00 ± 15.01		0.000
			P45	276.00 ± 19.27	180.0 ± 15.89		0.000
			P130[a]	287.78 ± 13.20	198.62 ± 9.68		0.000
	D	*Capacitance (pF)*	*P15*	68.70 ± 1.73	71.80 ± 3.15		0.698
			P21	105.00 ± 3.90	88.30 ± 2.86		0.001
			P35	111.00 ± 4.52	89.60 ± 4.60		0.001
			P45	109.00 ± 4.72	89.30 ± 5.80		0.002
			P130[a]	114.00 ± 4.91	100.00 ± 3.73		0.016
	E	*Input resistance hyperpolarized (Mohm)*	*P15*	106.50 ± 8.19	124.93 ± 8.03		0.029
			P21	58.71 ± 3.51	72.39 ± 3.54		0.016
			P35	46.90 ± 5.87	60.31 ± 5.60		0.087
			P45	46.59 ± 4.54	63.27 ± 5.44		0.014
			P130[a]	41.36 ± 3.20	54.87 ± 3.02		0.032
	F	*Input resistance depolarized (Mohm)*	*P15*	552.10 ± 66.34	467.30 ± 66.86		0.358
			P21	419.20 ± 40.55	535.00 ± 48.27		0.043
			P35	314.01 ± 58.72	472.30 ± 56.49		0.036
			P45	227.80 ± 41.71	430.50 ±64.52		0.007
			P130[a]	209.30 ± 20.99	352.40 ± 39.50		0.039
	H	*sEPSC frequency (Hz)*	*P15*	1.18 ± 0.09	1.45 ± 0.20		0.541
			P21	2.89 ± 0.19	2.37 ± 0.15		0.014
			P35	3.35 ± 0.19	2.38 ± 0.25		0.021
			P45	3.40 ± 0.20	2.50 ± 0.16		0.045
			P130[a]	3.86 ± 0.33	2.92 ± 0.23		0.000

a) Refers also to Mean and SEM in Figure 1

Supplementary Table 2. Developmental profile of the electrophysiological phenotypes (active properties) – refers to Figure 1 and 2 and S1

Figure		*Parameter*	*Age*	*Genotypes (Mean ± SEM)*		*Statistics*	
				WT	***LSL***	***Test***	***p value***
**Supplementary Figure 1**	**A**	*F-I slope (Hz/s)*	*P15*	*0.16 ± 0.007*	*43.08 ± 1.60*	*Post hoc Anova, LSD test*	*0.287*
			P21	*0.15 ± 0.006*	*39.16 ± 0.93*		*0.160*
			P35	*0.15 ± 0.005*	*42.53 ± 1.94*		*0.172*
			P45	*0.148 ± 0.007*	*39.76 ± 1.19*		*0.146*
			P130[a]	*0.16 ± 0.004*	*40.10 ± 1.94*		*0.735*
	**B**	*Maximum firing frequency (Hz)*	*P15*	*41.54 ± 1.83*	*43.08 ± 1.60*		*0.562*
			P21	*38.52 ± 1.21*	*39.16 ± 0.93*		*0.685*
			P35	*41.06 ± 1.20*	*42.53 ± 1.94*		*0.499*
			P45	*39.6 ± 1.4334*	*39.76 ± 1.19*		*0.937*
			P130[a]	*42.45 ± 1.1*	*40.10 ± 1.94*		*0.234*
	**C**	*AP threshold (mV)*	*P15*	*-43.05 ± 0.55*	*-43.08 ± 1.31*		*0.980*
			P21	*-39.24 ± 0.73*	*-40.32 ± 0.71*		*0.186*
			P35	*-42.32 ± 0.56*	*-42.96 ± 0.51*		*0.573*
			P45	*-41.30 ± 0.59*	*-41.72 ± 0.60*		*0.666*
			P130[a]	*-41.59 ± 0.52*	*-40.76 ± 0.51*		*0.381*

a) Refers also to Mean and SEM in Figure 1

Supplementary Table 3. Regression analysis of the developmental electrophysiological profile

Rheobase								
Model Summary		***R Square***	***F(1, 110)***	***Sig.***	***Constant***	***b1***	***b2***	***b3***
Equation[a]	**Linear**	0.203	28.089	0	181.169	0.865	*n.a.*	*n.a.*
	**Logarithmic**	0.335	55.335	0	-22.131	67.966	*n.a.*	*n.a.*
	**Inverse**	**0.442**	**87.084**	**0**	**327.689**	**-3109.781**	***n.a.***	***n.a.***
	**Quadratic**	0.409	37.697	0	80.47	5.141	-0.026	*n.a.*
	**Cubic**	0.465	31.322	0	-1.812	9.967	-0.091	0
	**Compound**	0.197	26.931	0	165.098	1.004	*n.a.*	*n.a.*
	**Power**	0.335	55.33	0	59.139	0.342	*n.a.*	*n.a.*
	**S**	0.467	96.216	0	5.853	-16.076	*n.a.*	*n.a.*
	**Growth**	0.197	26.931	0	5.107	0.004	*n.a.*	*n.a.*
	**Exponential**	0.197	26.931	0	165.098	0.004	*n.a.*	*n.a.*
	**Logistic**	0.197	26.931	0	0.006	0.996	*n.a.*	*n.a.*
Capacitance								
Model Summary		***R Square***	***F(1, 100)***	***Sig.***	***Constant***	***b1***	***b2***	***b3***
Equation[a]	**Linear**	0.058	6.14	0.015	97.647	0.123	*n.a.*	*n.a.*
	**Logarithmic**	0.14	16.308	0	60.712	11.795	*n.a.*	*n.a.*
	**Inverse**	**0.24**	**31.545**	**0**	**123.655**	**-611.015**	***n.a.***	***n.a.***
	**Quadratic**	0.304	21.637	0	67.656	1.374	-0.007	*n.a.*
	**Cubic**	0.307	14.472	0	62.752	1.66	-0.011	1.45E-05
	**Compound**	0.058	6.213	0.014	94.892	1.001	*n.a.*	*n.a.*
	**Power**	0.15	17.702	0	64.853	0.121	*n.a.*	*n.a.*
	**S**	0.27	37.009	0	4.822	-6.417	*n.a.*	*n.a.*
	**Growth**	0.058	6.213	0.014	4.553	0.001	*n.a.*	*n.a.*
	**Exponential**	0.058	6.213	0.014	94.892	0.001	*n.a.*	*n.a.*
	**Logistic**	0.058	6.213	0.014	0.011	0.999	*n.a.*	*n.a.*
Input resistance hyperpolarized								
Model Summary		***R Square***	***F(1, 94)***	***Sig.***	***Constant***	***b1***	***b2***	***b3***
Equation[a]	**Linear**	0.152	16.834	0	69.821	-0.232	*n.a.*	*n.a.*
	**Logarithmic**	0.283	37.074	0	129.339	-19.493	*n.a.*	*n.a.*
	**Inverse**	**0.43**	**70.993**	**0**	**27.085**	**951.31**	***n.a.***	***n.a.***
	**Quadratic**	0.348	24.84	0	100.744	-1.516	0.008	*n.a.*
	**Cubic**	0.43	23.103	0	131.859	-3.324	0.032	-9.03E-05
	**Compound**	0.169	19.052	0	63.3	0.996	*n.a.*	*n.a.*
	**Power**	0.304	40.996	0	168.621	-0.322	*n.a.*	*n.a.*
	**S**	0.433	71.688	0	3.454	15.212	*n.a.*	*n.a.*
	**Growth**	0.169	19.052	0	4.148	-0.004	*n.a.*	*n.a.*
	**Exponential**	0.169	19.052	0	63.3	-0.004	*n.a.*	*n.a.*
	**Logistic**	0.169	19.052	0	0.016	1.004	*n.a.*	*n.a.*
Input resistance depolarized								
Model Summary		***R Square***	***F(1, 107)***	***Sig.***	***Constant***	***b1***	***b2***	***b3***
Equation[a]	**Linear**	0.137	16.967	0	433.025	-1.823	*n.a.*	*n.a.*
	**Logarithmic**	0.207	27.849	0	841.182	-137.704	*n.a.*	*n.a.*

	**Inverse**	**0.255**	**36.538**	**0**	**139.753**	**6075.632**	***n.a.***	***n.a.***
	**Quadratic**	0.222	15.084	0	600.932	-8.918	0.042	*n.a.*
	**Cubic**	0.267	12.74	0	788.779	-19.924	0.193	-0.001
	**Compound**	0.147	18.438	0	360.306	0.995	*n.a.*	*n.a.*
	**Power**	0.233	32.574	0	1262.669	-0.421	*n.a.*	*n.a.*
	**S**	0.296	45.016	0	4.99	18.824	*n.a.*	*n.a.*
	**Growth**	0.147	18.438	0	5.887	-0.005	*n.a.*	*n.a.*
	**Exponential**	0.147	18.438	0	360.306	-0.005	*n.a.*	*n.a.*
	**Logistic**	0.147	18.438	0	0.003	1.005	*n.a.*	*n.a.*
sEPSCF frequency								
Model Summary		***R Square***	***F(1, 95)***	***Sig.***	***Constant***	***b1***	***b2***	***b3***
Equation [a]	**Linear**	0.239	29.808	0	2.376	0.016	*n.a.*	*n.a.*
	**Logarithmic**	0.319	44.509	0	-0.949	1.151	*n.a.*	*n.a.*
	**Inverse**	**0.362**	**53.888**	**0**	**4.841**	**-48.043**	***n.a.***	***n.a.***
	**Quadratic**	0.346	24.827	0	1.157	0.07	0	*n.a.*
	**Cubic**	0.346	16.393	0	1.066	0.075	0	2.84E-07
	**Compound**	0.194	22.849	0	2.123	1.005	*n.a.*	*n.a.*
	**Power**	0.288	38.408	0	0.655	0.402	*n.a.*	*n.a.*
	**S**	0.372	56.276	0	1.638	-17.908	*n.a.*	*n.a.*
	**Growth**	0.194	22.849	0	0.753	0.005	*n.a.*	*n.a.*
	**Exponential**	0.194	22.849	0	2.123	0.005	*n.a.*	*n.a.*
	**Logistic**	0.194	22.849	0	0.471	0.995	*n.a.*	*n.a.*
F-I Slope								
Model Summary		*R Square*	*F(1, 110)*	*Sig.*	*Constant*	*b1*	*b2*	*b3*
Equation [a]	**Linear**	0.017	1.904	0.17	0.14	7.48E-05	*n.a.*	*n.a.*
	**Logarithmic**	0.004	0.479	0.49	0.136	0.002	*n.a.*	*n.a.*
	**Inverse**	0	0.024	0.877	0.144	0.021	*n.a.*	*n.a.*
	**Quadratic**	0.049	2.81	0.065	0.152	0	3.03E-06	
	**Cubic**	0.059	2.256	0.086	0.162	-0.001	1.13E-05	-3.08E-08
	**Compound**	0.019	2.154	0.145	0.137	1.001	*n.a.*	*n.a.*
	**Power**	0.006	0.674	0.413	0.131	0.021	*n.a.*	*n.a.*
	**S**	0	0	0.995	-1.956	0.006	*n.a.*	*n.a.*
	**Growth**	0.019	2.154	0.145	-1.989	0.001	*n.a.*	*n.a.*
	**Exponential**	0.019	2.154	0.145	0.137	0.001	*n.a.*	*n.a.*
	**Logistic**	0.019	2.154	0.145	7.305	0.999	*n.a.*	*n.a.*
Maximum firing rate								
Model Summary		***R Square***	***F(1,110)***	***Sig.***	***Constant***	***b1***	***b2***	***b3***
Equation [a]	**Linear**	0.027	3.108	0.081	39.168	0.022	*n.a.*	*n.a.*
	**Logarithmic**	0.023	2.538	0.114	35.798	1.247	*n.a.*	*n.a.*
	**Inverse**	0.011	1.234	0.269	41.506	-34.836	*n.a.*	*n.a.*
	**Quadratic**	0.028	1.555	0.216	38.914	0.033	-6.48E-05	
	**Cubic**	0.034	1.265	0.29	40.845	-0.08	0.001	-5.76E-06
	**Compound**	0.029	3.342	0.07	38.616	1.001	*n.a.*	*n.a.*

	**Power**	0.025	2.835	0.095	35.251	0.034	*n.a.*	*n.a.*
	**S**	0.013	1.472	0.228	3.717	-0.967	*n.a.*	*n.a.*
	**Growth**	0.029	3.342	0.07	3.654	0.001	*n.a.*	*n.a.*
	**Exponential**	0.029	3.342	0.07	38.616	0.001	*n.a.*	*n.a.*
	**Logistic**	0.029	3.342	0.07	0.026	0.999	*n.a.*	*n.a.*
AP Threshold								
Model Summary		***R Square***	***F(1, 110)***	***Sig.***	***Constant***	***b1***	***b2***	***b3***
Equation [a]	***Linear***	*0.001*	*0.171*	*0.68*	*41.21*	*0.002*	*n.a.*	*n.a.*
	Logarithmic	*0*	*0.088*	*0.767*	*40.981*	*0.093*	*n.a.*	*n.a.*
	Inverse	*0*	*0.004*	*0.947*	*41.297*	*0.823*	*n.a.*	*n.a.*
	Quadratic	*0.002*	*0.244*	*0.784*	*41.544*	*-0.012*	*8.47E-05*	*n.a.*
	Cubic	*0.014*	*0.993*	*0.397*	*40.178*	*0.068*	*-0.001*	*4.27E-06*
	S	*0*	*0.029*	*0.865*	*3.72*	*-0.053*	*n.a.*	*n.a.*
	Growth	*0.002*	*0.338*	*0.562*	*3.714*	*7.71E-05*	*n.a.*	*n.a.*
	Exponential	*0.002*	*0.338*	*0.562*	*41.014*	*7.71E-05*	*n.a.*	*n.a.*
	Logistic	*0.002*	*0.338*	*0.562*	*0.024*	*1*	*n.a.*	*n.a.*
	Compound	*0.002*	*0.338*	*0.562*	*41.014*	*1*	*n.a.*	*n.a.*
	Power	*0.001*	*0.262*	*0.609*	*40.564*	*0.004*	*n.a.*	*n.a.*

a) Refers to the equations used in the different models:

Linear: F(postnatal day)= Constant + (b1 * postnatal day).

Logarithmic: F(postnatal day) = Constant + (b1 * ln(postnatal day))

Inverse: F(postnatal day) = Constant + (b1 / postnatal day)

Quadratic: F(postnatal day) = Constant + (b1 * postnatal day) + (b2 * postnatal day **2).

Cubic: F(postnatal day) = Constant + (b1 * postnatal day) + (b2 * postnatal day **2) + (b3 * postnatal day **3)

S-curve: F(postnatal day) = e**(Constant + (b1/ postnatal day))

Growth: F(postnatal day) = e**(Constant + (b1 * postnatal day))

Exponential: F(postnatal day) = Constant * (e**(b1 * postnatal day))

Logistic: F(postnatal day) = 1 / (1/u + (Constant * (b1** postnatal day)))

Compound: F(postnatal day) = Constant * (b1** postnatal day)

Power: F(postnatal day) = Constant * (postnatal day **b1)

Supplementary Table 4. Critical windows for the electrophysiological phenotype – refers to Figure 4

Panel	*Parameter*	*Ube3a manipulation*			*Mean ± SEM*		Statistics			
							**Two-way ANOVA (genotype X treatment)** ***F/p values***	***Post-hoc*** **Bonferroni p values**		
			Age	***Treatment***	Genotype			**LSL-VEH vs. WT-VEH**	**LSL-VEH vs WT-TAM**	**LSL-VEH vs LSL-TAM**
					WT	***LSL***				
E	***Rheobase (pA)***	***reinstatement***	***P21***	***VEH***	*261.54 ± 21.12*	*196.37 ± 10.76*	*F(1,108) = 4.17, p = 0.044*	0.041	0.006	0.001
				TAM	*264.00 ± 13.34*	*257.50 ± 10.41*				
L			***P70***	***VEH***	*304.12 ± 13.3*	*218.82 ± 15.06*	*F(1,67) = 0.12, p = 0.76*	0.009	0.004	1
				TAM	*260.77 ± 23.66*	*210.66 ± 17.24*				
					WT	***Flox***				
S		***deletion***	***P45***	***VEH***	*260.00 ± 10.10*	*275.56 ± 13.1*	*F(1,69) = 0.59, p = 0.49*			
				TAM	*280.56 ± 14.78*	*249.42 ± 13.8*				
					WT	***LSL***				
F	***Input Resistance (Mohm)***	***reinstatement***	***P21***	***VEH***	*162.17 ± 17.77*	*304.14 ± 22.77*	*F(1,108) = 6.27, p = 0.014*	0.003	0.0001	0.001
				TAM	*185.58 ± 22.2*	*220.32 ± 25.53*				
M			***P70***	***VEH***	*187.42 ± 17.18*	*349.00 ± 36.15*	*(F(1,67) = 0.04, p = 0.16*	0.005	0.004	1
				TAM	*234.20 ± 19.75*	*292.70 ± 24.8*				
					WT	***Flox***				
T		***deletion***	***P45***	***VEH***	*147.46 ± 12.35*	*121.60 ± 9.16*	*F(1,69) = 1.99 , p = 0.026*			
				TAM	*118.02 ± 11.55*	*151.13 ± 15.35*				
					WT	***LSL***				
G	***sEPSC frequency (Hz)***	***reinstatement***	***P21***	***VEH***	*4.70 ± 0.70*	*3.29 ± 0.25*	*F(1,107) = 5.16,*	0.016	0.3	0.3
				TAM	*4.01 ± 0.35*	*4.02 ± 0.24*				
N			***P70***	***VEH***	*4.88 ± 0.38*	*3.08 ± 0.26*	*F(1,87) = 0.22, p = 0.63*	0.002	0.017	1
				TAM	*4.46 ± 0.31*	*3.02 ± 0.34*				
					WT	*Flox*				
U		***deletion***	***P45***	***VEH***	*5.36 ± 0.33*	*5.25 ± 0.43*	*F(1,69) = 0.22, p = 0.63*			
				TAM	*4.60 ± 0.32*	*4.38 ± 0.34*				
